# Measuring Food Literacy: Progressing the Development of an International Food Literacy Survey Using a Content Validity Study

**DOI:** 10.3390/ijerph18031141

**Published:** 2021-01-28

**Authors:** Donna Fingland, Courtney Thompson, Helen Anna Vidgen

**Affiliations:** School of Exercise and Nutrition Sciences, Queensland University of Technology, Victoria Park Road, Kelvin Grove, QLD 4059, Australia; donna.fingland@health.qld.gov.au (D.F.); c21.thompson@hdr.qut.edu.au (C.T.)

**Keywords:** food literacy, measurement, questionnaire, validation, survey, assessment tool, expert consensus, content validity

## Abstract

*Background*: The term “food literacy” is increasingly used to describe the knowledge, skills and behaviours needed to meet food needs. The aim of this research was to determine content validity for an International Food Literacy Survey. *Methods*: The literature was searched for existing items to form an item pool to measure the eleven components of food literacy. Expert consensus was investigated through two related online surveys. Round 1 participants were researchers who had been involved in the development of a food literacy measure (*n* = 18). Round 2 participants were authors of papers who had used the term (*n* = 85). Level of agreement was determined quantitatively using the Content Validity Index and compared to open ended qualitative comments. *Results*: Consensus was achieved on 119 items. Components varied in the ease with which existing validated items could be found and the number of items achieving consensus. Items related to food prepared within the home were more likely to achieve consensus. Additional issues included limited shared understanding of the scope of the term, the validity of items varying according to context and a limited health focus. *Conclusions*: This study provides a valuable basis upon which to progress the development of a measure.

## 1. Introduction

Nutritionists have long had an interest in how people go about feeding themselves [1,2,3]. Over time, various elements have been identified and measured, reflective of dominant ideals and contemporary eating. They have included cooking [4], nutrition knowledge [5,6] food agency [7], food involvement [8], food wellbeing [9] and food skills [10]. These elements, particularly nutrition knowledge [11,12,13,14] and cooking skills [15,16,17], are also the focus of interventions. Evidence to support the effectiveness of these interventions [18,19] or their impact on dietary intake [20] is not strong. Food literacy is an emerging term which attempts to more comprehensively describe the knowledge, skills and behaviours used to meet food needs, inclusive of these elements, in an effort to better reflect contemporary food and eating [21]. The widespread use of this term has spanned continents, and reflects a diverse range of food related outcomes from chronic disease risk, to lifeskills, ecological sustainability and economic grow [22,23,24,25,26,27].

The first published definition appeared in 2001, however there have been several since then [28]. A scoping review in 2017 identified thirty-eight unique definitions [29]. The most widely cited definition is that developed by Vidgen and Gallegos [30]. The definition states “food literacy is a collection of inter-related knowledge, skills and behaviours required to plan, manage, select, prepare and eat food to meet needs and determine intake. It is the scaffolding that empowers individuals, households, communities or nations to protect diet quality through change and strengthen dietary resilience over time” [21]. This conceptualisation of food literacy is made up of eleven components which fall into four domains of: (i) planning and management, (ii) selection, (iii) preparation and (iv) eating [21]. The authors highlight that the enactment of these interdependent components are likely to be highly contextual. The relative importance of each will change over time, in response to dynamic micro, meso and macro environments ranging from changes in family structure to global food systems. These components are common to most other definitions and conceptualisations of food literacy. Variation in definitions are predominantly regarding the extent to which additional components such as food systems knowledge is considered and the emphasis on nutrition [29,31,32,33].

A recent scoping review of food literacy measures identified twelve tools including five that were explicitly developed to measure the construct [34]. These were in Italy [35], Switzerland [36], Australia [37], the US [7] and The Netherlands [23] and focused on their respective adult populations. All except the Swiss tool were based on the Vidgen and Gallegos food literacy definition and conceptual framework. The Swiss Short Food Literacy Questionnaire was developed based on a nutrition-specific health literacy framework [36]. Despite agreement on the overarching framework for the remaining tools, the items within them varied markedly, indicative of the contexts and outcomes for which they were developed. The diversity of these tools reflects the complexity of measuring food literacy and the challenge in gaining consensus to allow comparative measurement. Given the term food literacy is used globally and applied in a broad range of contexts, it would be beneficial to have a tool which can measure across these.

A valid and reliable food literacy measure is needed to: (1) assess and monitor food literacy in individuals, communities and populations; (2) better target interventions to improve food literacy; (3) enable evaluation of these interventions, and (4) increase the understanding of the relationship between food literacy and dietary intake [29,38,39]. The current absence of an international and comparable scale to measure food literacy limits evidence based advancement of this field. The purpose of this study was to progress the development of an international measure of food literacy by undertaking the first step of a content validity study. To control for the variation in conceptualisations of food literacy, the Vidgen model was chosen as it is the most used, and successive reviews have concluded that it represents an agreed minimum set of components of food literacy.The study aimed to gain consensus from food literacy experts for the proposed items for an International Food Literacy Survey to establish content validity.

## 2. Materials and Methods

This content validity study had three stages. First, the literature was searched to find existing items to generate an item pool to be put forward for expert consensus. Expert consensus then took place in two rounds. The first round involved researchers who had been involved in the development of a food literacy measure based on the Vidgen definition, to capture those who had in depth knowledge of the components and had deliberated alignment of items with them. This was to determine if there is conceptual consensus from those involved in developing tools, on the inclusion of items in an International Food Literacy Survey and the alignment of these items with each component. Results from this round were then analysed to develop a revised list of items for presentation in round two. Participants in round two were a larger set of experts made up of those who had cited the Vidgen definition, to capture the broader application of the measure consensus across a range of contexts.

### 2.1. Survey Development

The academic literature was searched for measures that assessed the individual components of food literacy [21]. Items were then pooled from these identified pre-validated assessment tools. The aim was to pool a significantly greater number of items than required for the final survey to enhance the content validation process. The pooled items were sorted for relevance and mapped to the eleven components of the food literacy model [21]. This was done in consultation with Vidgen and Gallegos to ensure items collectively represented the full meaning of each component.

Items assessing intention or confidence were excluded where an equivalent item measuring knowledge, skills or behaviour was available [21]. Intention to eat healthy food and confidence in ones cooking skills is also often overreported due to social desirability bias [40,41]. Items that did not represent the model, duplicates and items from non-validated measures were excluded. As the Italian and Dutch tools were developed on the same conceptual framework all of their items were included.

The remaining items were mapped along the continuum of knowledge, attitude and behaviour to assess balance across all areas. Finally, further searching was undertaken to identify items for components with a limited number pooled from the initial search. Items were unchanged from the original source. Where sufficient items could not be located, new items were developed by the researchers. New items focused on the aspect of the component that was not addressed by existing items and were framed to measure knowledge, skills or behaviour using the language convention of included items where possible.

In both rounds the experts were asked to rate the content relevance of each item. A Likert scale was used for research participants to rate each item: 1 = the item is not relevant, 2 = the item is somewhat relevant, 3 = the item is quite relevant, 4 = the item is highly relevant. Participants also had the opportunity to provide open ended comments for each item and regarding any aspects of the component not covered by the item pool, and if there were any alternative items they felt would assess each component better. It was not compulsory for participants to respond to all items.

### 2.2. Data Collection

Items were grouped under their respective component of food literacy which sat within one of the four domains of planning and management, selection, preparation and eating. The survey was conducted using Key Survey a secure online platform which allows for deidentified participation [42]. The order of the eleven food literacy components (and associated items) were randomised. This aimed to minimise an uneven response rate across the components due to potential non-completion of the survey due to fatigue. Round two results were gathered within three months of round one.

### 2.3. Data Analysis

A content validity index (CVI) was calculated for each item using the equation below [43,44,45]:

(1) $$CVI=\frac{Number\;of\;experts\;that\;rated\;the\;item\;3\;or\;4\;for\;relevance}{Total\;number\;of\;experts\;that\;rated\;the\;item},$$

Round one data was analysed according to the CVI for each item and related open-ended comments. Items with a CVI ≥ 0.71 were included in the round two survey. These items were reviewed alongside their open-ended comments. These results were reviewed independently by the first and senior author. This resulted in items being re-categorised to different components, being reworded, additional items being sourced to more fully describe the component or the item being discarded. Round two data was again analysed according to CVI and a thematic analysis of all comments independently by the first and second authors. Themes and indicative quotes were reviewed by the senior author. Items with a score ≥ 0.71 were considered to have achieved consensus [43].

### 2.4. Participant Identification

Round one research participants comprised of food literacy coalition members who are active researchers within Australia, Italy, The Netherlands and Canada. These members have been involved in the development and validation of food literacy assessment tools in their respective countries or have actively progressed the food literacy knowledge base. Twenty-nine food literacy experts were invited to participate from Canada (*n* = 9) The Netherlands (*n* = 7), Italy (*n* = 7), and Australia (*n* = 6).

For round two all authors of all papers that had cited the Vidgen and Gallegos model [21] (96 published articles, 259 authors) at the time of the study (March 2018), excluding those who were sampled in round one, were invited to participate. An additional six researchers who had directly contacted Vidgen to collaborate in advancing the food literacy knowledge base but had not yet published in the field were also invited. Email addresses for participants were sourced from publicly available information such as their cited article or https://www.researchgate.net. Those for whom an email address could not be sourced (*n* = 19) or were not able to be contacted as they had retired from their position (*n* = 2), were excluded. This resulted in 244 participants invited to participate from 28 countries (Table 1). The adequacy of the sample was determined by extent to which participants were representative of the range of studies (round one) and papers (round two) citing the model. No additional characteristics of participants e.g., age, gender, sector, were collected.

Methods to increase response rates included personalised emails, inclusion of the citing papers title within the email to increase relevance, and weekly reminder emails throughout the survey completion period [46]. All subjects gave their informed consent for inclusion before they participated in the study. The study was conducted in accordance with the Declaration of Helsinki, and the protocol was approved by the Ethics Committee of the Queensland University of Technology (1800000173).

## 3. Results

### 3.1. Participants

Eighteen experts (response rate 62%) participated in round one of the content validity study. Participants represented each of the invited countries i.e., Canada (*n* = 6), Australia (*n* = 5), The Netherlands (*n* = 4) and Italy (*n* = 3). As it was not compulsory to complete all items the number of experts who provided content validation varied between items. The average item completion rate was 15 participants (range 12–18).

Round two of the content validity study yielded a participation rate of 35% (*n* = 85). The contributing experts included authors representing 51% (*n* = 49) of the 96 articles invited and 71% (*n* = 20) of the 28 countries invited (Table 1). The average item completion rate was 63 participants (range 48–84).

### 3.2. Content Validity Results

Initially pooled items assessed a mix of knowledge, skills and behaviour or perceived skills, confidence, attitude and intention. Items measuring the latter were frequently sourced from nutrition education interventions [47,48]. In contrast, questions measuring current knowledge, skills and behaviour often stemmed from consumer research and cross-sectional studies examining associations between food-related behaviours and health outcomes [2,3,49,50]. The round one survey contained 229 items originating from 45 existing tools [2,3,4,15,23,35,47,48,49,50,51,52,53,54,55,56,57,58,59,60,61,62,63,64,65,66,67,68,69,70,71,72,73,74,75,76,77,78,79,80,81,82,83,84,85,86] and five newly developed items. Table 2 outlines the number of items mapped to each of the eleven components of food literacy. The categorisation of each item in rounds one and two, their source and level of consensus is presented in the Supplementary Material.

Components varied in the ease with which existing validated items could be found. Many items could be found that aligned with Component 3.1. *Make a good tasting meal from whatever is available. This includes being able to prepare commonly available foods, efficiently use common pieces of kitchen equipment and having a sufficient repertoire of skills to adapt recipes (written or unwritten) to experiment with food and ingredients*. Few could be found for Component 2.3 *Judge the quality of food*. This was then reflected in the number of items achieving consensus, with Component 2.3 having only three items with a CVI of at least 0.71.

Of the 229 items within the round one survey, 48% (*n* = 111) achieved a CVI score of at least 0.71 (Table 2). Key issues identified by participants when completing the survey were disagreement with the categorisation of items against the component; similar items not being grouped together which made it difficult to discern between them; a failure to address all aspects of the component by the items provided and the high number of items leading to participant fatigue.

The survey was revised for round two, specifically, items with scores below 0.71 were removed, items were re-categorised to improve alignment with components, similar items within a component were grouped to facilitate assessment of comparable items and new items for missing aspects were sourced or developed. The round two survey contained 151 items (Table 2). Overall, twenty questions moved components between round one and round two (refer to Table 2 and Supplementary Material). This was predominantly between components 4.2 and 1.3. Twenty-six new items were developed for round two in response to participant feedback that the items presented did not fully capture the meaning of the term (refer to Supplementary Material). These were predominantly in components 1.3 and 4.3. Components 3.1 and 3.2 in the preparation domain, required no re-categorisation or development of new items.

Items relating to the knowledge, skills and behaviours required to meet immediate food needs e.g., “how often do you plan meals ahead” scored higher than those which were more critical and systems focused e.g., “how concerned are you about the routine use of animal antibiotics to promote growth of farm animals”. Similarly, items which focused on food preparation, grocery shopping and formal meal times, scored higher than items regarding other ways and places people get their food from.

Following the second round, a CVI score ≥0.71 was reached on 79% (*n* = 119) from the initial 151 items (Table 2). These items are presented in Table 3. The number of items is inconsistently distributed across components (range 3–33, median 9). This was not always indicative of the total number of items originally presented. Analysis of open-ended comments provide insights into this inconsistency. Items with the highest level of consensus did not tend to come from tools explicitly developed to measure food literacy. Items that focused on individuals or households scored highly. Items related to communities or national food issues did not reach the threshold level of consensus.

### 3.3. Thematic Analysis of Open Ended Comments in Round Two

Analysis of the comments for round two identified the following key themes. Quotes included cannot be attributed as all responses were non-identifiable. The data however can identify each quote originating from a unique respondent.

#### 3.3.1. The Field of Food Literacy May Be too New to Achieve Content Validity

Despite the Vidgen definition being widely used and cited by all participants, there was inadequate understanding of the meaning of the individual components. This can be summarised by the following statements from participants:

*“You need to build a nomothetic net of constructs first—people to understand what constructs measuring are before items can be developed to assess them.”*

*“I think what you are measuring needs to be more accurately defined before these items are judged for suitability.”*

Participants also commented on the lack of a definition for certain components. For example, the component ‘Judge the quality of food’ received questions concerning the meaning of ‘quality’. Comments included:

*“depends on how you determine quality—local, nutritious, expensive?”*

*“What is the meaning of quality? That word may mean different things to each person (e.g., healthy, organic, fresh, brand name, sensorial quality, nutritional quality, organic, no additives?”*

Respondents identified the subjective nature of the following terms within items as particularly problematic: “ethical practices”, “stressed”, “diet quality”, “best quality”, “budget”, “staple foods”, “processed foods” and “expectations”.

#### 3.3.2. The Validity of Items Varied According to Context

Experts noted other contributing factors to food choice were not considered. For example, the item, ‘I am able to buy healthy foods for my family on a budget’ (CVI score 0.82) received the comment:

*“This could be very context based. For example, if someone is living somewhere quite remote and there is little to no access to healthy foods then the budget doesn’t matter.”*

Certain items within the component 1.2 Plan food intake (formally and informally) so that food can be regularly accessed through some source, irrespective of changes in circumstances or environment were also identified as problematic as they failed to consider contextual factors. Comments received included:

*“Have to take contextual factors into account—e.g., not having a fridge, or having a food market close by, will make a lot of difference to planning meals. Plus, do we want to take into account the very large number of poor people who produce their own food?”*

*“You are only looking at food access from a health lens and perspective and missing the systems elements which affect the availability of healthy foods.”*

Participants commented that proposed items measuring Components 1.1 *Prioritise money and time for food* and 1.3 *Make feasible food decisions which balance food needs with available resources* would result in people who are food insecure or are of a low socio-economic status receiving a low food literacy score. Comments included:

*“I think the topic of this question is very important but that the wording may be flawed. For those that just don’t have money to feed themselves and their families, setting aside money is not an option.”*

*“I think this could easily create shame for someone who can’t invest in their food due to a significant cost of living and low/fixed income”*

*“Ability and knowledge or interest are not always the same thing for low-income families. I think this comes back to the question of whether someone can be highly knowledgeable and engaged but not able, due to economic circumstances.”*

Adaptability across contexts was particularly noted for different countries and cultures. Measuring the ability to access foods from multiple stores or restaurants was noted as not relevant to a person who grows their own food or accesses food from emergency food programs. Similarly, questions pertaining to the use of kitchen equipment may not apply to people without electricity or cultures using traditional cooking methods. 

The items assessing knowledge around appropriate intake of foods and nutrients experts commented will also vary between countries. For example, an appropriate intake of types of fat will be different between cultures, beliefs of health professionals and requirements of different disease states. Likewise, the notion of eating socially (Component 4.3) is culturally defined.

#### 3.3.3. Items Tend to be Health Focused

Another theme identified from the study participants was whether the tool should be assessing food literacy for a health outcome or food literacy for a more holistic range of outcomes. Comments included:

*“Whether you are assessing food literacy or healthy food literacy needs to be considered, and all sections need to be considered in light of this as currently the questions are very much focused on health. Different people will focus on health as much as they can within their means”*

*“There seems to be an assumption built into the questions, but not evidenced in the definition of the component, that people are planning around HEALTH rather than around other considerations, like taste, cultural relevance, etc. Either the questions referencing health need to be reworded, or the component needs to be rewritten—otherwise, I don’t think it’s reasonable to say that the questions measure the component.”*

*“healthy food’ is not a stated component of the construct”*

*“The reliance on ’healthy’ as a proxy for a lot of assumptions in terms of the types of meals prepared could get you into some trouble.”*

A lack of a definition for ‘healthy foods’ was also raised as a shortcoming.

## 4. Discussion

The aim of this study was to progress the development of the International Food Literacy Survey through expert consensus for the proposed items aligned to Vidgen and Gallegos’ eleven components of food literacy [21]. The findings of this study highlight that while there appears to be consensus on a definition, identifying items to measure the full meaning of the term across contexts is challenging.

The term has emerged to more comprehensively describe the knowledge, skills and behaviours used to meet food needs in our contemporary food system than more established constructs such as cooking and nutrition knowledge. Experts however, were more able to agree on these constructs than on a more modern imagining of eaters. Items regarding food preparation, grocery shopping and structured meals were more easily sourced from the literature and more consistently understood by participants. Items related to eating outside the home and the planning and selection involved in this were less consistently understood. Given the increase in the contribution of food prepared and consumed outside the home to total dietary intake and diet quality throughout the world, this is an important gap [87,88,89,90,91]. Additionally, food literacy is considered to apply to all eaters, not just dietary gatekeepers responsible for household food provisioning, as such, its measurement should not be overly focused on these skills. Dietary gatekeepers are typically women, with this role often detracting from their capacity to be involved in paid work, therefore a continued focus on these elements is likely to re-enforce the status quo of gendered food roles.

The complexity of developing a tool for use across socioeconomic levels emerged from the qualitative feedback. For example, participants commented that some items within the tool did not consider people who are unable to prioritise food due to other priorities such as housing or medical expenses. Items assessing the ability to plan ahead are not relevant to people who do not have refrigeration or make food choices daily from their crops or local markets. Likewise, factors such as access to foods, eating socially and living alone need to be reviewed to reflect different living situations. The Vidgen model was designed to be applicable across socio-economic contexts as the empirical research from which it was derived specifically focused on people experiencing disadvantage [21]. The challenge exists therefore in the identification of items that do not stigmatise respondants but rather provide a mechanism to highlight socio-economic status as a determinant of food intake independent of food literacy.

Items assessing nutrition knowledge and cooking methods require adaptation to be understood internationally and across cultures. Authors of tools developed in Italy, The Netherlands and Switzerland did not experience these issues as their study populations were residents within their respective countries [23,35,36]. These studies did highlight however the diversity in tools across populations. Despite being based on the same conceptual framework, the Dutch and Italian tools contain very different items reflective of their individual populations and cultures [23,35].

Experts also requested clarification around whether the tool is assessing food literacy to support health or to more holistically meet food needs. Published tools have incorporated a focus on health including the Self Perceived Food Literacy Scale [23] and the Short Food Literacy Questionnaire [36]. Palumbo identified food literacy as the ability of someone to make health enhancing food choices [22]. Vidgen’s model identifies multiple potential outcomes of food literacy including health, diet quality, food security, social connectedness and ethical food choices [92]. Truman, Lane and Elliot found very few definitions focused on health with critical knowledge a more common outcome of interest [29]. One of the opportunities presented by the broad adoption of the term “food literacy” across sectors, is that its component parts may describe the knowledge, skills and behaviours for a more holistic view of eating well. There were few items, however, reflective of healthy sustainable eating within global food systems, that achieved consensus.

A recent review of food literacy measures recognised that despite having a common theoretical underpinning, items differed widely depending upon the intended purpose of the measure [34]. The Italian food literacy tool, for example, includes items focused on the food system more broadly, the interaction between the individual and the food environment [35]. This is consistent with the Vidgen conceptualisation of food literacy existing at individual, household, community and national levels. Items which reflected this broader set of knowledge, skills and behaviours however, scored poorly. This may be because these concepts are more abstract. High scoring items tended to come from tools which examined cooking, food preparation, family meal times and shopping practices [2,4] which are constructs which have been measured for much longer and participants are therefore more likely to have a clear understanding and conceptualisation of. It may also be because they represent more immediate, functional needs, with broader constructs seen as less essential for meeting immediate food needs. Various authors have applied the functional, interactive and critical continuums of health literacy to food literacy [24,93]. Informed by the development of the European Health Literacy Survey Questionnaire, this framework was applied to item development by the Italian and Dutch researchers however, these items failed to gain consensus from the participants in this study [23,35,94]. It may also be that the full meaning of these questions was impacted in their translation to English.

A key strength of this study was the participation of a broad range of international experts in the field of food literacy. Involvement of experts is an important element in establishing content validity of a new tool. This method enabled input from experts from geographically diverse locations. Anonymous online feedback enabled contribution without bias from other participants and avoided dominant points of view. Inviting a different expert panel for each round reduced the risk of participant fatigue and attrition [95]. A further strength is that the large majority of items were obtained from previously validated measures. All items also assessed one conceptualisation of food literacy as identified by Vidgen & Gallegos. This was advantageous in specifying a consistent conceptualisation of the construct but may have also been a limitation in that it represented a less contemporary impression of the term. Items were taken directly from their source and therefore included a mix of statements and phrases which may have impacted on responses.

A further limitation of the study includes the time to complete the survey which reduced the number of participants completing it in its entirety. Whilst both rounds in this study requested experts identify additional items, due to time and participant fatigue, this seldom occurred. A further limitation was lack of clarity and agreement for the components of food literacy from the round two experts who, despite citing the definition, may not have had a knowledge of the components. This impacted the ability to obtain consensus for the items. Since this study was conducted in early 2018, several more measures of food literacy have been developed. This would have contributed to an expanded item pool.

## 5. Conclusions

The complexity of developing an International Food Literacy Survey is highlighted by this study. This is due to an inconsistent understanding of the term amplified by the diversity of contexts, e.g., different countries, cultures, socioeconomic levels, across which it would be enacted, etc. To enable the progression of the development of the International Food Literacy Survey, further work is needed to gain a shared understanding. This will enable identification of items relevant to a broad range of cultures and economic levels and assist in exploring other aspects of measurement such as the description of levels and results. A tool could be then trialled across contexts to determine its transferability and application. Eaters should also be involved in the validation of items, as experts in their own lives and how they navigate their food environment to meet needs.

## Figures and Tables

**Table 1:** Invited and participated experts for round two by country

Country	Invited (*n*)	Participated (*n*)
Australia	53	18
Canada	47	16
USA	38	12
UK	16	10
Iran	10	4
France	9	1
Switzerland	9	2
Spain	8	2
Finland	7	2
Slovenia	6	2
Brazil	5	3
Italy	5	3
Belgium	5	2
New Zealand	4	0
Germany	3	0
Turkey	2	2
Hong Kong	2	1
The Netherlands	2	1
Norway	2	0
Ireland	2	0
Mexico	2	0
Thailand	1	1
Andorra	1	1
Lebanon	1	1
Indonesia	1	1
Malta	1	0
Denmark	1	0
Japan	1	0
**Total**	**244**	**85**

**Table 2:** The total number of items presented under each food literacy component in Round One and Round Two Surveys and the number (%) achieving consensus i.e., CVI score ≥ 0.71. (CVI scores of individual items is presented in the Supplementary Material).

		Round 1		Round 2	
		Total	Consensus	Total	Consensus
1. Plan and manage food	1.1 Prioritise money and time for food ^1^	20	10 (50)	8	7 (88)
	1.2 Plan food intake (formally and informally) so that food can be regularly accessed through some source, irrespective of changes in circumstances or environment	15	13 (87)	16	11 (69)
	1.3 Make feasible food decisions which balance food needs (e.g., nutrition, taste, hunger) with available resources (time, money, skills, equipment) ^2^	28	11 (39)	12	9 (75)
2. Select food	2.1 Access food through multiple sources and know the advantages and disadvantages of these ^3^	16	4 (25)	6	3 (50)
	2.2 Determine what is in a food product, where it came from, how to store it and use it	21	9 (43)	12	11 (92)
	2.3 Judge the quality of food ^4^	17	3 (18)	6	5 (83)
3. Prepare food	3.1 Make a good tasting meal from whatever is available. This includes being able to prepare commonly available foods, efficiently use common pieces of kitchen equipment and having a sufficient repertoire of skills to adapt recipes (written or unwritten) to experiment with food and ingredients	27	20 (74)	42	33 (79)
	3.2 Apply basic principles of safe food hygiene and handling	32	9 (28)	11	11 (100)
4. Eat food	4.1 Understand food has an impact of personal wellbeing ^5^	23	7 (30)	8	6 (75)
	4.2 Demonstrate self-awareness of the need to personally balance food intake. This includes knowing foods to include for good health, foods to restrict for good health and appropriate portion size and frequency ^6^	15	12 (80)	11	9 (82)
	4.3 Join in and eat in a social way	15	13 (87)	19	14 (74)
	TOTAL	229	111 (48)	151	119 (79)

^1^ Six items were recategorized to a different component following round 1: 1 to 1.2; 2 to 1.3; 3 to 3.1; ^2^ Three items that were recategorized to a different component following round 1: 1 to 1.2; 2 to 4.2; ^3^ Four items were recategorized to a different component following round 1: 1 to 1.3; 2 to 2.2.; 1 to 2.3; ^4^ One item was recategorized to a different component following round 1: 1 to 2.2; ^5^ Two items were recategorized to a different component following round 1: 2 to 4.2; ^6^ Four items were recategorized to a different component following round 1: 4 to 1.2.

**Table 3:** International Food Literacy Survey items within each component achieving consensus (ie CVI score ≥ 0.71) in Round two of the Content Validity Study.

Item	Source
**Component 1.1. Prioritise time and money for food**	
Compared with other daily decisions, my food choices are not very important.	[53]
Eating healthy meals just takes too much time.	[52]
I always make sure I have enough money set aside to feed myself even if I have limited money.	Developed
I often eat unhealthy foods because I’ve run out of time to buy or prepare something better.	Developed
Meals are an important part of the day for me/my household.	[2]
I really care what I eat.	[80]
Food shopping takes up too much of my time.	[50]
**Component 1.2 Plan food intake (formally and informally) so that food can be regularly accessed through some source irrespective of changes in circumstances or environments**	
How often do you plan meals ahead? (e.g., for the day/week ahead)	[4]
How often do you prepare meals in advance? e.g., packed lunch, partly preparing a meal in advance	[4]
I am able to buy healthy foods for my family on a budget.	[75]
I write a shopping list to take with me when I shop for food.	[2]
Do you keep basic items in your cupboard for putting meals together? e.g., herbs/spices, dried/tinned goods?	[4]
I know how to budget for groceries/food.	[71]
Should my circumstances change during the day e.g., I have to work late, one of my children get sick, we have extra people coming over for dinner; I can easily change my plans so that I still eat a healthy meal.	Developed
How often do you shop with specific meals in mind?	[4]
How often do you plan how much food to buy?	[4]
Do you bring healthy snacks for yourself when you are on the go? For example, fruit, cherry tomatoes, nuts.	[23]
Are you able to eat healthy when you feel stressed?	[23]
**Component 1.3 Make feasible food decisions which balance food needs (eg nutrition, taste, hunger) with available resources (eg.time, money, skills, equipment)**	
Do you purchase healthy food, even if you have limited money? For example, vegetables, fruit or whole grain products	[23]
Is eating food that is healthy an important influencer when deciding what to buy to eat outside the home?	[73]
Do you purchase healthy foods, even if it is a bit more expensive? For example, vegetables, fruit or whole grain products	[23]
How often do you compare prices before you buy food?	[4]
I know how much money I spend on food from one week to the next.	Developed
I have good intentions about healthy eating but I find it difficult to actually eat that way.	Developed
How often do you adjust meals to include specific ingredients that are more “budget friendly,” like on sale or in your refrigerator or pantry?	[75]
It is important to me that the food I eat on a typical day is easy to prepare.	[78]
I always try to get the best quality for the best price.	[77]
**Component 2.1 Access food through multiple sources and know the advantages and disadvantages of these**	
I compare prices between product variants in order to get the best value food.	[3]
I balance my intake of foods from restaurants, cafes, fast food outlets and what I make at home so that I can still have a healthy diet overall.	Developed
I look for store specials and plan to take advantage of them when I go shopping.	[75]
**Component 2.2 Determine what is in a food product, where it came from, how to store it and how to use it**	
Do you use the nutritional label on food products to guide your purchases?	[35]
Do you compare the calories, fat, sugar or salt content of different products?	[23]
To me product information is of high importance. I need to know what the product contains.	[75]
How easy do you believe it is to find information about the nutritional properties of the foods you plan to buy?	[35]
Do you read the storage and use-by information on food packets?	[4]
I know the environmental impact of my eating habits.	Developed
How easy do you believe it is to find information about how to prepare the food that you purchased?	[3]
I understand the ethical practices behind how the food I eat was produced.	Developed
Is the impact on the community where food comes from an important influencer on your choice of foods?	[73]
I can make an accurate judgement on the nutritional content of the food I’ve selected.	Developed
How often do you pay attention to the country of origin indications on food products you buy?	[58]
**2.3 Judge the quality of food**	
I can accurately judge the quality of fresh food so that it meets my expectations.	Developed
Do you read the best-before date on food?	[4]
I can accurately judge the quality of food I eat when I am out (restaurant, café) so that it meets my expectations.	Developed
Do you buy food in season to save money?	[4]
I can accurately judge the quality of processed or convenience food so that it meets my expectations.	Developed
**3.1 Make a good tasting meal from whatever food is available. This includes being able to prepare commonly available foods, efficiently use common pieces of kitchen equipment and having a sufficient repertoire of skills to adapt recipes (written or unwritten) to experiment with food or ingredients**	
When lacking ingredients, do you find it easy to make changes to recipes without compromising the results?	[35]
How would you rate your skills in being able to cook from basic ingredients?	[51]
Are you able to cook from basic ingredients?	[65]
I am able to cook healthy foods for my family on a budget.	[75]
Are you able to prepare fresh vegetables in different ways? For example, cooking, steaming or stir frying or in different dishes	[23]
I am able to prepare a meal without a recipe.	[66]
How confident are you at following recipes when cooking?	[4]
I feel I don’t have the skills to cook (healthy) meals for my family.	[86]
How good are you at each cooking task?	
1. Handling pans on a stove	[81]
2. Using an oven	[81]
3. Steaming food (where the food doesn’t touch the water but gets cooked by the steam)	[4]
4. Boiling or simmering food (cooking it in a pan of hot, boiling/bubbling water)	[4]
5. Stewing food (cooking it for a long time (usually more than an hour) in a liquid or sauce at a medium heat, not boiling) e.g., beef stew	[4]
6. Roasting food in the oven, for example raw meat/chicken, fish, vegetables etc.	[4]
7. Frying/stir-frying/sautéing food in a frying pan/wok with oil or fat using the hob/gas rings/hot plates	[4]
8. Microwaving food (not drinks/liquid) including heating ready-meals	[4]
9. Cooking a piece of raw or frozen meat/chicken/fish, (not processed or partially-prepared)	[48]
10. Choosing a spice or herb that goes well with the food I am cooking	[48]
Do you use leftovers to create another meal?	[4]
I have a lot of knowledge about how to cook using different methods.	[55]
Are you able to prepare fresh fish in different ways? For example, grilling, pan frying or stewing, or in different dishes	[23]
How would you rate your skills in planning a quick, healthy meal using only foods already in your home, and then preparing these foods so they can be served all together within 1 hour or less?	[48]
Are you able to prepare a meal using fresh ingredients? So, without pre-packed and processed foods?	[23]
How would you rate your skills in coordinating the preparation and cooking of a few food dishes at the same time so you can serve them all together for a meal?	[48]
Knowing how to cook is important to me.	[71]
How good are you at each preparation task?	[4]
1. Using knife skills in the kitchen	[60]
2. Using measuring cups and spoons	[81]
6. Chopping, mixing and stirring foods, for example chopping vegetables, dicing an onion, cubing meat, mixing and stirring food together in a pot/bowl	[4]
7. Peeling, chopping or slicing vegetables or fruit	[48]
8. Freezing vegetables or fruit, from raw to bagged in my home freezer	[48]
I do not like to cook because it takes too much time.	[60]
**3.2 Apply basic principles of safe hygiene and handling**	
Do you wash your hands before you start handling food?	[68]
Do you wash your hands after handling raw meat, poultry or fish?	[68]
Do you use the same knife for cutting raw and then cooked meat?	[68]
Do you wash fruit and vegetables that don’t need to be peeled before eating them?	[51]
I clean the kitchen surfaces before meals are prepared.	[76]
I know how to store meats and dairy.	[71]
I clean the kitchen surfaces before and after meals are prepared.	[76]
Do you follow the instructions for storage on packaged foods?	[51]
Once I unfreeze meat, I don’t refreeze it again.	[76]
I know how to store perishable foods (fruits, vegetables, meats, dairy).	[71]
I keep raw and cooked meals separated.	[76]
**4.1. Understand food has an impact on personal wellbeing**	
I understand what foods to eat to prevent diet related chronic disease (such as heart disease).	Developed
The types of food I eat affects how I feel.	[52]
The types of food I eat affects my health.	[52]
The types of food I eat affects my weight.	[52]
How easy do you believe it is to find information on foods for the prevention or management of a disease or condition?	[35]
How easy do you believe it is to use the food/nutritional recommendations to improve your state of health?	[35]
**4.2 Demonstrate self-awareness of the need to personally balance food intake. This includes knowing foods to include for good health, food to restrict for good health, and appropriate portion size and frequency**	
I think about nutrition when I choose what I eat.	[79]
I make a conscious effort to try to follow a healthy diet.	[69]
Do health professionals recommend that people should be eating more, the same amount, or less of the following foods? 1. Fruit 2. Food and drinks with added sugar 3. Vegetables 4. Fatty foods 5. Processed red meat 6. Wholegrain 7. Salty foods 8. Water	[70]
How many servings of fruit and vegetables per day do you think you should eat as a minimum? (One serving could be, for example, an apple or a handful of chopped carrots) 2 3 4 5 or more Not sure	[70]
I know what foods to eat to keep me healthy.	Developed
When deciding what to feed your family, how often do you think about healthy food choices?	[61]
Which of these types of fats do health professionals recommend that people should eat less of? Unsaturated fats, trans fat, saturated fats 1 Not sure 2 Eat less 3 Not eat less	[70]
Do you balance meals based on nutrition advice on what is healthy?	[4]
How easy do you believe it is to understand the food/nutritional recommendations provided by your doctor or dietitian?	[35]
**4.3 Join in and eat in a social way**	
How often do you eat together at home with others?	[15]
Mealtime is a time for talking with others, it is about more than just eating food.	[84]
I find that dining with friends is an important part of my social life.	[75]
Do you find it important to eat at the dinner table if you are eating with others?	[23]
I eat on the go.	[2]
Are you involved in other activities while eating? For example, reading, working, using ipad/iphone or watching television?	[23]
Eating brings people together in an enjoyable way.	[74]
My eating habits and behaviours make it hard for me to join in with others when I’m eating.	Developed
I sit at a table to eat my meals.	Developed
In my household people often have their meals at different times.	[72]
It is important that I eat at least one meal a day with others.	[84]
I eat meals/snacks while working or studying.	[2]
Do you find it important to eat dinner at the same time if you are with others?	[23]
I prefer eating with people than alone.	Developed

## Data Availability

The data presented in this study are available in the Supplementary Material for study two and is available upon request from the corresponding author for study one.

## Supplementary Materials

The following are available online at https://www.mdpi.com/1660-4601/18/3/1141/s1, Table S1: The categorisation, level of agreement and source of each item presented in Rounds one and two of the International Food Literacy Survey Content Validity Study.

## References

[1] Bisogni C.A., Jastran M., Shen L., Devine C.M. (2005). A Biographical Study of Food Choice Capacity: Standards, Circumstances, and Food Management Skills. J. Nutr. Educ. Behav..

[2] Crawford D., Ball K., Mishra G., Salmon J., Timperio A. (2007). Which food-related behaviours are associated with healthier intakes of fruits and vegetables among women?. Public Health Nutr..

[3] Grunert K.G., Brunsø K., Bisp S. (1993). Food-Related Life Style: Development of a Cross-Culturally Valid Instrument for Market Surveillance.

[4] Lavelle F., McGowan L., Hollywood L., Surgenor D., McCloat A., Mooney E., Caraher M., Raats M., Dean M. (2017). The development and validation of measures to assess cooking skills and food skills. Int. J. Behav. Nutr. Phys. Act..

[5] Worsley A. (2002). Nutrition knowledge and food consumption: Can nutrition knowledge change food behaviour?. Asia Pac. J. Clin. Nutr..

[6] Parmenter K., Wardle J. (1999). Development of a general nutrition knowledge questionnaire for adults. Eur. J. Clin. Nutr..

[7] Lahne J., Wolfson J.A., Trubek A. (2017). Development of the Cooking and Food Provisioning Action Scale (CAFPAS): A new measurement tool for individual cooking practice. Food Qual. Prefer..

[8] Marshall D., Bell R. (2004). Relating the food involvement scale to demographic variables, food choice and other constructs. Food Qual. Prefer..

[9] Block L.G., Grier S.A., Childers T.L., Davis B., Ebert J.E.J., Kumanyika S., Laczniak R.N., Machin J.E., Motley C.M., Peracchio L. (2011). From Nutrients to Nurturance: A Conceptual Introduction to Food Well-Being. J. Public Policy Mark..

[10] Fordyce-Voorham S. (2011). Identification of Essential Food Skills for Skill-based Healthful Eating Programs in Secondary Schools. J. Nutr. Educ. Behav..

[11] Pollard C.M., Miller M.R., Daly A.M., Crouchley K.E., O’Donoghue K.J., Lang A.J., Binns C.W. (2008). Increasing fruit and vegetable consumption: Success of the Western Australian Go for 2&5^®^ campaign. Public Health Nutr..

[12] Trieu K., McMahon E., Santos J.A., Bauman A., Jolly K., Bolam B., Webster J. (2017). Review of behaviour change interventions to reduce population salt intake. Int. J. Behav. Nutr. Phys. Act..

[13] Kansagra S.M., Kennelly M.O., Nonas C.A., Curtis C.J., Van Wye G., Goodman A., Farley T.A. (2015). Reducing sugary drink consumption: New York City’s approach. Am. J. Public Health.

[14] Maes L., Van Cauwenberghe E., Van Lippevelde W., Spittaels H., De Pauw E., Oppert J.-M., Van Lenthe F.J., Brug J., De Bourdeaudhuij I. (2012). Effectiveness of workplace interventions in Europe promoting healthy eating: A systematic review. Eur. J. Public Health.

[15] Herbert J., Flego A., Gibbs L., Waters E., Swinburn B., Reynolds J., Moodie M. (2014). Wider impacts of a 10-week community cooking skills program—Jamie’s Ministry of Food, Australia. BMC Public Health.

[16] Garcia A.L., Reardon R., McDonald M., Vargas-Garcia E.J. (2016). Community Interventions to Improve Cooking Skills and Their Effects on Confidence and Eating Behaviour. Curr. Nutr. Rep..

[17] Block K., Gibbs L., Staiger P.K., Gold L., Johnson B., Macfarlane S., Long C., Townsend M. (2012). Growing Community: The Impact of the Stephanie Alexander Kitchen Garden Program on the Social and Learning Environment in Primary Schools. Health Educ. Behav..

[18] Reicks M., Kocher M., Reeder J. (2018). Impact of Cooking and Home Food Preparation Interventions Among Adults: A Systematic Review (2011–2016). J. Nutr. Educ. Behav..

[19] Caraher M., Seeley A. (2010). Cooking in schools: Lessons from the UK. J. Home Econ. Inst. Aust..

[20] Clifford Astbury C., Penney T.L., Adams J. (2019). Comparison of individuals with low versus high consumption of home-prepared food in a group with universally high dietary quality: A cross-sectional analysis of the UK National Diet & Nutrition Survey (2008–2016). Int. J. Behav. Nutr. Phys. Act..

[21] Vidgen H.A., Gallegos D. (2014). Defining food literacy and its components. Appetite.

[22] Palumbo R. (2016). Sustainability of well-being through literacy. The effects of food literacy on sustainability of well-being. Agric. Agric. Sci. Procedia.

[23] Poelman M.P., Dijkstra S.C., Sponselee H., Kamphuis C.B.M., Battjes-Fries M.C.E., Gillebaart M., Seidell J.C. (2018). Towards the measurement of food literacy with respect to healthy eating: The development and validation of the self perceived food literacy scale among an adult sample in the Netherlands. Int. J. Behav. Nutr. Phys. Act..

[24] Benn J. (2014). Food, nutrition or cooking literacy-a review of concepts and competencies regarding food education. Int. J. Home Econ..

[25] Sausheva O.S., Lizina O.M., Ilyakova I.E. (2019). Food Literacy as a Component of a Person’s Economic Security. Religacion.

[26] Rose N., Lourival I. (2019). Hegemony, Counter-Hegemony and Food Systems Literacy: Transforming the Global Industrial Food System. Aust. J. Environ. Educ..

[27] FAO, United Arab Emirates University (2019). Stepping up School-Based Food and Nutrition Education: Exploring Challenges, Finding Solutions and Building Partnerships.

[28] Kolasa M.K., Peery G.A., Harris G.N., Shovelin G.K. (2001). Food Literacy Partners Program: A Strategy To Increase Community Food Literacy. Top. Clin. Nutr..

[29] Truman E., Lane D., Elliott C. (2017). Defining food literacy: A scoping review. Appetite.

[30] Vidgen H.A. (2014). Food Literacy: What Is It and Does It Influence What We Eat?. Ph.D. Thesis.

[31] Azevedo Perry E., Thomas H., Samra H.R., Edmonstone S., Davidson L., Faulkner A., Petermann L., Manafò E., Kirkpatrick S.I. (2017). Identifying attributes of food literacy: A scoping review. Public Health Nutr..

[32] Yuen E.Y.N., Thomson M., Gardiner H. (2018). Measuring Nutrition and Food Literacy in Adults: A Systematic Review and Appraisal of Existing Measurement Tools. Health Lit. Res. Pract..

[33] Krause C., Sommerhalder K., Beer-Borst S., Abel T. (2018). Just a subtle difference? Findings from a systematic review on definitions of nutrition literacy and food literacy. Health Promot. Int..

[34] Amouzandeh C., Fingland D., Vidgen H.A. (2019). A Scoping Review of the Validity, Reliability and Conceptual Alignment of Food Literacy Measures for Adults. Nutrients.

[35] Palumbo R., Annarumma C., Adinolfi P., Vezzosi S., Troiano E., Catinello G., Manna R. (2017). Crafting and applying a tool to assess food literacy: Findings from a pilot study. Trends Food Sci. Technol..

[36] Krause C.G., Beer-Borst S., Sommerhalder K., Hayoz S., Abel T. (2018). A short food literacy questionnaire (SFLQ) for adults: Findings from a Swiss validation study. Appetite.

[37] Begley A., Paynter E., Dhaliwal S.S. (2018). Evaluation Tool Development for Food Literacy Programs. Nutrients.

[38] McKecknie R., Vidgen H. (2016). Measuring food literacy. Food Literacy: Key Concepts for Health and Education.

[39] Truman E., Elliott C. (2018). Barriers to Food Literacy: A Conceptual Model to Explore Factors Inhibiting Proficiency. J. Nutr. Educ. Behav..

[40] Slater J.J., Mudryj A.N. (2016). Self-perceived eating habits and food skills of Canadians. J. Nutr. Educ. Behav..

[41] Adams J., Goffe L., Adamson A.J., Halligan J., O’Brien N., Purves R., Stead M., Stocken D., White M. (2015). Prevalence and socio-demographic correlates of cooking skills in UK adults: Cross-sectional analysis of data from the UK National Diet and Nutrition Survey. Int. J. Behav. Nutr. Phys. Act..

[42] Queensland University of Technology Key Survey at QUT. https://survey.qut.edu.au/site/.

[43] Lynn R.M. (1986). Determination and Quantification Of Content Validity. Nurs. Res..

[44] Wynd C.A., Schmidt B., Schaefer M.A. (2003). Two quantitative approaches for estimating content validity. West J. Nurs. Res..

[45] Rattray J., Jones M.C. (2007). Essential elements of questionnaire design and development. J. Clin. Nurs..

[46] Anseel F., Lievens F., Schollaert E., Choragwicka B. (2010). Response rates in organizational science, 1995-2008: A meta-analytic review and guidelines for survey researchers. J. Bus. Psychol..

[47] Wallace R., Lo J., Devine A. (2016). Tailored nutrition education in the elderly can lead to sustained dietary behaviour change. J. Nutr. Health Ageing.

[48] Thomas H.M., Irwin J.D. (2011). Cook It Up! A community-based cooking program for at-risk youth: Overview of a food literacy intervention. BMC Res. Notes.

[49] Ailawadi K.L., Neslin S.A., Gedenk K. (2001). Pursuing the Value-Conscious Consumer: Store Brands versus National Brand Promotions. J. Mark..

[50] Buckley M., Cowan C., McCarthy M. (2007). The convenience food market in Great Britain: Convenience food lifestyle (CFL) segments. Appetite.

[51] Barton K.L., Wrieden W.L., Anderson A.S. (2011). Validity and reliability of a short questionnaire for assessing the impact of cooking skills interventions. J. Hum. Nutr. Diet..

[52] Bauer K.W., Larson N.I., Nelson M.C., Story M., Neumark-Sztainer D. (2009). Socio-environmental, personal and behavioural predictors of fast-food intake among adolescents. Public Health Nutr..

[53] Bell R., Marshall D.W. (2003). The construct of food involvement in behavioral research: Scale development and validation. Appetite.

[54] Brissette I., Lowenfels A., Noble C., Spicer D. (2013). Predictors of total calories purchased at fast-food restaurants: Restaurant characteristics, calorie awareness, and use of calorie information. J. Nutr. Educ. Behav..

[55] Burton M., Reid M., Worsley A., Mavondo F. (2017). Food skills confidence and household gatekeepers‘ dietary practices. Appetite.

[56] Byrd-Bredbenner C. (2005). Food Preparation Knowledge and Confidence of Young Adults. J. Nutr. Recipe Menu Dev..

[57] Byrd-Bredbenner C., Wheatley V., Schaffner D., Bruhn C., Blalock L., Maurer J. (2007). Development and implementation of a food safety knowledge instrument: Food science education research. J. Food Sci. Educ..

[58] Cerjak M., Haas R., Brunner F., Tomic M. (2014). What motivates consumers to buy traditional food products? Evidence from Croatia and Austria using word association and laddering interviews. Br. Food J..

[59] Chamhuri N., Batt P.J. (2015). Consumer perceptions of food quality in Malaysia. Br. Food J..

[60] Condrasky M.D., Williams J.E., Catalano P.M., Griffin S.F. (2011). Development of psychosocial scales for evaluating the impact of a culinary nutrition education program on cooking and healthful eating. J. Nutr. Educ. Behav..

[61] Dollahite J.S., Pijai E.I., Scott-Pierce M., Parker C., Trochim W. (2014). A Randomized Controlled Trial of a Community-Based Nutrition Education Program for Low-Income Parents. J. Nutr. Educ. Behav..

[62] Dunn C., Jayaratne K.S.U., Baughman K., Levine K. (2014). Teaching Basic Cooking Skills: Evaluation of the North Carolina Extension “Cook Smart, Eat Smart” Program. J. Fam. Consum. Sci..

[63] Flego A., Herbert J., Gibbs L., Swinburn B., Keating C., Waters E., Moodie M. (2013). Methods for the evaluation of the Jamie Oliver Ministry of Food program, Australia. BMC Public Health.

[64] Food and Drug Administration (2016). 2014 FDA Health and Diet Survey.

[65] Garcia A.L., Vargas E., Lam P.S., Shennan D.B., Smith F., Parrett A. (2014). Evaluation of a cooking skills programme in parents of young children—A longitudinal study. Public Health Nutr..

[66] Hartmann C., Dohle S., Siegrist M. (2013). Importance of cooking skills for balanced food choices. Appetite.

[67] Hwang J., Cranage D. (2010). Customer health perceptions of selected fast-food restaurants according to their nutritional knowledge and health consciousness. J. Foodserv. Bus. Res..

[68] Jevšnik M., Hlebec V., Raspor P. (2008). Consumers’ awareness of food safety from shopping to eating. Food Control.

[69] Kearney J.M., Gibney M.J., Livingstone B.E., Robson P.J., Kiely M., Harrington K. (2001). Attitudes towards and beliefs about nutrition and health among a random sample of adults in the Republic of Ireland and Northern Ireland. Public Health Nutr..

[70] Kliemann N., Wardle J., Johnson F., Croker H. (2016). Reliability and validity of a revised version of the General Nutrition Knowledge Questionnaire. Eur. J. Clin. Nutr..

[71] Levy J., Auld G. (2004). Cooking Classes Outperform Cooking Demonstrations for College Sophomores. J. Nutr. Educ. Behav..

[72] Mallinson L.J., Russell J.M., Barker M.E. (2016). Attitudes and behaviour towards convenience food and food waste in the United Kingdom. Appetite.

[73] NatCen Social Research British Social Attitudes Survey. http://bsa.natcen.ac.uk/media/38990/bsa-26-annotated-questionnaires-2008.pdf.

[74] Neumark-Sztainer D., Larson N.I., Fulkerson J.A., Eisenberg M.E., Story M. (2010). Family meals and adolescents: What have we learned from Project EAT (Eating Among Teens)?. Public Health Nutr..

[75] Pinard C.A., Uvena L.M., Quam J.B., Smith T.M., Yaroch A.L. (2015). Development and testing of a revised Cooking Matters for Adults Survey. Am. J. Health Behav..

[76] Sanlier N., Konaklioglu E. (2012). Food safety knowledge, attitude and food handling practices of students. Br. Food J..

[77] Scholderer J., Brunsø K., Bredahl L., Grunert K.G. (2004). Cross-cultural validity of the food-related lifestyles instrument (FRL) within Western Europe. Appetite.

[78] Steptoe A., Pollard T.M., Wardle J. (1995). Development of a Measure of the Motives Underlying the Selection of Food: The Food Choice Questionnaire. Appetite.

[79] Stotts J.L., Lohse B. (2007). Reliability of the ecSatter Inventory as a Tool to Measure Eating Competence. J. Nutr. Educ. Behav..

[80] The Health and Social Care Information Centre Health Survey for England 2007, Healthy Lifestyles: Knowledge, Attitudes and Behaviour. http://digital.nhs.uk/catalogue/PUB00415.

[81] Thonney P.F., Bisogni C.A. (2006). Cooking Up Fun! A Youth Development Strategy that Promotes Independent Food Skills. J. Nutr. Educ. Behav..

[82] Vilaro M.J., Zhou W., Colby S.E., Byrd-Bredbenner C., Riggsbee K., Olfert M.D., Barnett T.E., Mathews A.E. (2017). Development and Preliminary Testing of the Food Choice Priorities Survey (FCPS): Assessing the Importance of Multiple Factors on College Students’ Food Choices. Eval. Health Prof..

[83] Winkler E., Turrell G. (2010). Confidence to Cook Vegetables and the Buying Habits of Australian Households. J. Am. Diet. Assoc..

[84] Woodruff S.J., Kirby A.R. (2013). The associations among family meal frequency, food preparation frequency, self-efficacy for cooking, and food preparation techniques in children and adolescents. J. Nutr. Educ. Behav..

[85] Worsley A., Wang W.C., Burton M. (2015). Food concerns and support for environmental food policies and purchasing. Appetite.

[86] Ramirez E. (2015). Development and Implementation of the Generations Eating Together through Cooking (G.E.T.T. Cooking) Curriculum and Its Effects on an Inter-Generational Population: A Pilot Study. Diploma Thesis.

[87] Andrade G.C., Da Costa Louzada M.L., Azeredo C.M., Ricardo C.Z., Martins A.P.B., Levy R.B. (2018). Out-of-Home Food Consumers in Brazil: What do They Eat?. Nutrients.

[88] Guthrie J.F., Lin B.-H., Frazao E.J. (2002). Role of food prepared away from home in the American diet, 1977–1978 versus 1994–1996: Changes and consequences. J. Nutr. Educ. Behav..

[89] Kearney J., Hulshof K., Gibney M.J. (2001). Eating patterns–temporal distribution, converging and diverging foods, meals eaten inside and outside of the home–implications for developing FBDG. Public Health Nutr..

[90] Zang J., Luo B., Wang Y., Zhu Z., Wang Z., He X., Wang W., Guo Y., Chen X., Wang C. (2018). Eating Out-of-Home in Adult Residents in Shanghai and the Nutritional Differences among Dining Places. Nutrients.

[91] Wellard-Cole L., Jung J., Kay J., Rangan A., Chapman K., Watson W.L., Hughes C., Ni Mhurchu C., Bauman A., Gemming L. (2018). Examining the Frequency and Contribution of Foods Eaten Away From Home in the Diets of 18- to 30-Year-Old Australians Using Smartphone Dietary Assessment (MYMeals): Protocol for a Cross-Sectional Study. JMIR Res. Protoc..

[92] Vidgen H. (2016). Relating Food Literacy to Nutrient and Health.

[93] Cullen T., Hatch J., Martin W., Higgins J., Sheppard R. (2015). Food Literacy: Definition and Framework for Action. Can. J. Diet. Pract. Res..

[94] Sorensen K., Van den Broucke S., Pelikan J.M., Fullam J., Doyle G., Slonska Z., Kondilis B., Stoffels V., Osborne R.H., Brand H. (2013). Measuring health literacy in populations: Illuminating the design and development process of the European Health Literacy Survey Questionnaire (HLS-EU-Q). BMC Public Health.

[95] Keeney S., Hasson F., McKenna H.P., Wiley I. (2011). The Delphi Technique.

