# Supplementary material for: Measuring Food Literacy: Progressing the Development of an International Food Literacy Survey Using a Content Validity Study
Source: Int J Environ Res Public Health. 2021 Jan 28;18(3):1141. doi: 10.3390/ijerph18031141 (PMC7908320; doi:10.3390/ijerph18031141)
Supplement: Supplementary file 1 [file ijerph-18-01141-s001.pdf]

Table S1: The categorisation, level of agreement and source of each item presented in Rounds one and two of the International Food Literacy Survey Content Validity Study

Item	source	Round one[1]			Round two		
		component	score	cvi	component	score	CVI
COMPONENT 1.1. PRIORITISE TIME AND MONEY FOR FOOD							
Compared with other daily decisions, my food choices are not very important. 1 Strongly disagree 2 Disagree 3 Neutral 4 Agree 5 Strongly agree	(Bell & Marshall, 2003)	1.1	14/16	0.88	1.1	65/78	0.83
Eating healthy meals just takes too much time. 1 Strongly disagree 2 Disagree 3 Neutral 4 Agree 5 Strongly agree	(Bauer, Larson, Nelson, Story, & Neumark-Sztainer, 2009)	1.1	12/16	0.75	1.1	63/76	0.83
I always make sure I have enough money set aside to feed myself even if I have limited money	Developed				1.1	61/76	0.80
I often eat unhealthy foods because I've run out of time to buy or prepare something better	Developed				1.1	62/78	0.79
Meals are an important part of the day for me/my household. 1 Never 2 Rarely 3 Sometimes 4 Usually 5 Always	(Crawford, Ball, Mishra, Salmon, & Timperio, 2007)	1.1	15/16	0.94	1.1	60/77	0.78
I really care what I eat. 1 Strongly disagree 2 Disagree 3 Neutral 4 Agree 5 Strongly agree	(The Health and Social Care Information Centre, 2009)	1.1	12/16	0.75	1.1	59/77	0.77
Food shopping takes up too much of my time.	(Buckley, Cowan, & McCarthy, 2007)	1.1	9/13	0.69	1.1	57/77	0.74
I often skip meals because I've run out of time to buy or prepare food	Developed				1.1	52/76	0.68
I don't think about food much each day. 1 Strongly disagree 2 Disagree 3 Neutral 4 Agree 5 Strongly agree	(Bell & Marshall, 2003)	1.1	11/16	0.69			
It is too much work to cook. 1 Strongly disagree 2 Disagree 3 Neutral 4 Agree 5 Strongly agree	(Condrasky, Williams, Catalano, & Griffin, 2011)	1.1	11/16	0.69			
How often do you find cooking a meal a chore? 1 Never 2 Rarely 3 Sometimes 4 Usually 5 Always	(Crawford, Ball, Mishra, Salmon, & Timperio, 2007)	1.1	10/16	0.63			
Cooking needs to be planned in advance. 1 Strongly disagree 2 Disagree 3 Neutral 4 Agree 5 Strongly agree	(Grunert, Brunsø, & Bisp, 1993)	1.1	9/15	0.60			
Healthy foods are enjoyable. 1 Strongly disagree 2 Disagree 3 Neutral 4 Agree 5 Strongly agree	(The Health and Social Care Information Centre, 2009)	1.1	9/15	0.60			
I do food shopping whenever I can fit it into my routine. 1 Strongly disagree 2 Disagree 3 Neutral 4 Agree 5 Strongly agree	(Crawford, Ball, Mishra, Salmon, & Timperio, 2007)	1.1	7/15	0.47			
Talking about what I ate or am going to eat is something I like to do. 1 Strongly disagree 2 Disagree 3 Neutral 4 Agree 5 Strongly agree	(Bell & Marshall, 2003)	1.1	7/16	0.44			
I try to do my food shopping as quickly as possible. 1 Strongly disagree 2 Disagree 3 Neutral 4 Agree 5 Strongly agree	(Buckley, Cowan, & McCarthy, 2007)	1.1	5/16	0.31			
Healthy eating is just another fad. 1 Strongly disagree 2 Disagree 3 Neutral 4 Agree 5 Strongly agree	(The Health and Social Care Information Centre, 2009)	1.1	5/16	0.31			
COMPONENT 1.2 PLAN FOOD INTAKE (FORMALLY AND INFORMALLY) SO THAT FOOD CAN BE REGULARLY ACCESSED THROUGH SOME SOURCE IRRESPECTIVE OF CHANGES IN CIRCUMSTANCES OR ENVIRONMENTS							
a) How often do you plan meals ahead? (e.g. for the day/week ahead)	(Lavelle et al., 2017)	1.2	11/12	0.92	1.2	60/67	0.90
How often do you prepare meals in advance? e.g. packed lunch, partly preparing a meal in advance. 1 Never 2 Rarely 3 Sometimes 4 Usually 5 Always	(Lavelle et al., 2017)	1.2	12/13	0.92	1.2	59/71	0.83
I am able to buy healthy foods for my family on a budget. 1 Strongly disagree 2 Disagree 3 Neutral 4 Agree 5 Strongly agree	(Pinard, Uvena, Quam, Smith, & Yaroch, 2015)	1.3	12/13	0.92	1.2	56/68	0.82

Item	source	Round one[1]			Round two		
		component	score	cvi	component	score	CVI
a) I write a shopping list to take with me when I shop for food.	(Crawford, Ball, Mishra, Salmon, & Timperio, 2007)	1.2	12/13	0.92	1.2	57/70	0.81
Do you keep basic items in your cupboard for putting meals together? e.g. herbs/spices, dried/tinned goods? 1 Never 2 Rarely 3 Sometimes 4 Usually 5 Always	(Lavelle et al., 2017)	1.2	11/14	0.79	1.2	56/69	0.81
I know how to budget for groceries/food. 1 Strongly disagree 2 Disagree 3 Neutral 4 Agree 5 Strongly agree	(Levy & Auld, 2004)	1.1	12/14	0.86	1.2	55/69	0.80
Should my circumstances change during the day e.g. I have to work late, one of my children get sick, we have extra people coming over for dinner; I can easily change my plans so that I still eat a healthy meal. 1 Never 2 Rarely 3 Sometimes 4 Usually 5 Always	Developed				1.2	51/68	0.75
How often do you shop with specific meals in mind? 1 Never 2 Rarely 3 Sometimes 4 Usually 5 Always	(Lavelle et al., 2017)	1.2	9/12	0.79	1.2	52/70	0.74
How often do you plan how much food to buy? 1 Never 2 Rarely 3 Sometimes 4 Usually 5 Always	(Lavelle et al., 2017)	1.2	10/14	0.71	1.2	51/71	0.72
Do you bring healthy snacks for yourself when you are on the go? For example, fruit, cherry tomatoes, nuts 1 Never 2 Rarely 3 Sometimes 4 Usually 5 Always	(Poelman et al., 2018)	4.2	10/13	0.77	1.2	48/67	0.72
Are you able to eat healthy when you feel stressed? 1 Never 2 Rarely 3 Sometimes 4 Usually 5 Always	(Poelman et al., 2018)	4.2	10/13	0.77	1.2	49/69	0.71
If you have something to eat, do you reflect on what you have eaten before on that day? 1 Never 2 Rarely 3 Sometimes 4 Usually 5 Always	(Poelman et al., 2018)	4.2	10/13	0.77	1.2	47/69	0.68
Do you have healthy snacks for yourself in stock? For example nuts, carrots, cherry tomatoes, or mini cucumbers 1 Never 2 Rarely 3 Sometimes 4 Usually 5 Always	(Poelman et al., 2018) et al	1.2	12/14	0.86	1.2	47/71	0.66
If you have something to eat, do you consider what you will eat later that day? 1 Never 2 Rarely 3 Sometimes 4 Usually 5 Always	(Poelman et al., 2018)	4.2	9/12	0.75	1.2	45/69	0.65
I am able to maintain the diet quality of the foods I eat over time. 1 Strongly disagree 2 Disagree 3 Neutral 4 Agree 5 Strongly agree	Developed				1.2	41/63	0.65
If I had to relocate to a new and unfamiliar area it would take me a while to get into a routine to feed myself to my satisfaction. 1 Strongly disagree 2 Disagree 3 Neutral 4 Agree 5 Strongly agree	Developed				1.2	32/68	0.47
I always plan what we are going to eat a couple of days in advance. 1 Strongly disagree 2 Disagree 3 Neutral 4 Agree 5 Strongly agree	(Scholderer, Brunsø, Bredahl, & Grunert, 2004)	1.2	12/14	0.86			
Usually I do not decide what to buy until I am in the shop. (I have a tendency to buy a few more things than I had planned) 1 Strongly disagree 2 Disagree 3 Neutral 4 Agree 5 Strongly agree	(Scholderer, Brunsø, Bredahl, & Grunert, 2004)	1.2	12/14	0.86			
About how many times per week do you eat a packed lunch taken from home to work/place of study? (Crawford et al., 2007) 1 Never 2 Less than once a week 3 1-3 times a week 4 4-6 times a week 5 Daily	(Crawford, Ball, Mishra, Salmon, & Timperio, 2007)	1.2	11/14	0.79			
a) How often do you decide on the night what you will eat for dinner that night?	(Crawford, Ball, Mishra, Salmon, & Timperio, 2007)	1.2	9/12	0.75			
How often do you prepare or cook dishes ahead of time to eat through the week? 1 Never 2 Rarely 3 Sometimes 4 Usually 5 Always	(Crawford, Ball, Mishra, Salmon, & Timperio, 2007)	1.2	10/14	0.71			
About how many times per week do you eat meals that are prepared/cooked and eaten at home? 1 Never 2 Less than once a week 3 1-3 times a week 4 4-6 times a week 5 Daily	(Crawford, Ball, Mishra, Salmon, & Timperio, 2007)	1.2	10/14	0.71			
How often do you know or plan in the morning what you will eat for dinner that night? 1 Never 2 Rarely 3 Sometimes 4 Usually 5 Always	(Crawford, Ball, Mishra, Salmon, & Timperio, 2007)	1.2	9/14	0.64			
How often do you know or plan the day or night before what you will eat for lunch the next day? 1 Never 2 Rarely 3 Sometimes 4 Usually 5 Always	(Crawford, Ball, Mishra, Salmon, & Timperio, 2007)	1.2	9/14	0.64			
COMPONENT 1.3 MAKE FEASIBLE FOOD DECISIONS WHICH BALANCE FOOD NEEDS (EG NUTRITION, TASTE, HUNGER) WITH AVAILABLE RESOURCES (EG.TIME, MONEY, SKILLS, EQUIPMENT)							

Item	source	Round one[1]			Round two		
		component	score	cvi	component	score	CVI
Do you purchase healthy food, even if you have limited money? For example, vegetables, fruit or whole grain products 1 Never 2 Rarely 3 Sometimes 4 Usually 5 Always	(Poelman et al., 2018)	1.1	13/16	0.81	1.3	52/60	0.87
Is eating food that is healthy an important influencer when deciding what to buy to eat outside the home? 1 Never 2 Rarely 3 Sometimes 4 Usually 5 Always	(NatCen Social Research, 2008)	2.1	11/15	0.73	1.3	57/67	0.85
Do you purchase healthy foods, even if it is a bit more expensive? For example, vegetables, fruit or whole grain products 1 Never 2 Rarely 3 Sometimes 4 Usually 5 Always	(Poelman et al., 2018)	1.1	12/15	0.80	1.3	55/69	0.80
How often do you compare prices before you buy food? 1 Never 2 Rarely 3 Sometimes 4 Usually 5 Always	(Lavelle et al., 2017)	1.3	10/14	0.71	1.3	54/69	0.78
I know how much money I spend on food from one week to the next. 1 Strongly disagree 2 Disagree 3 Neutral 4 Agree 5 Strongly agree	Developed				1.3	54/69	0.78
I have good intentions about healthy eating but I find it difficult to actually eat that way. 1 Strongly disagree 2 Disagree 3 Neutral 4 Agree 5 Strongly agree	Developed				1.3	51/66	0.77
How often do you adjust meals to include specific ingredients that are more "budget friendly," like on sale or in your refrigerator or pantry? 1 Never 2 Rarely 3 Sometimes 4 Usually 5 Always	(Pinard, Uvena, Quam, Smith, & Yaroch, 2015)	1.3	11/13	0.85	1.3	51/68	0.75
It is important to me that the food I eat on a typical day is easy to prepare. 1 Strongly disagree 2 Disagree 3 Neutral 4 Agree 5 Strongly agree	(Steptoe, Pollard, & Wardle, 1995)	1.3	11/14	0.79	1.3	50/69	0.72
I always try to get the best quality for the best price. 1 Strongly disagree 2 Disagree 3 Neutral 4 Agree 5 Strongly agree	(Scholderer, Brunsø, Bredahl, & Grunert, 2004)	1.3	12/14	0.86	1.3	50/70	0.71
I know the general price of staple foods. 1 Strongly disagree 2 Disagree 3 Neutral 4 Agree 5 Strongly agree	Developed				1.3	37/67	0.55
I know how long it takes me to shop for food each week. 1 Strongly disagree 2 Disagree 3 Neutral 4 Agree 5 Strongly agree	Developed				1.3	36/68	0.53
When I prepare a meal, it always takes me much longer than I thought it would. 1 Strongly disagree 2 Disagree 3 Neutral 4 Agree 5 Strongly agree	Developed				1.3	31/66	0.47
How good are you at knowing what budget you have to spend on food? 1 Very Limited 2 Limited 3 Basic 4 Good 5 Very good	(Lavelle et al., 2017)	1.3	12/14	0.86			
Do you balance meals based on nutrition advice on what is healthy? 1 Never 2 Rarely 3 Sometimes 4 Usually 5 Always	(Lavelle et al., 2017)	1.3	10/14	0.71			
I compare prices of at least a few brands before I choose one. 1 Strongly disagree 2 Disagree 3 Neutral 4 Agree 5 Strongly agree	(Ailawadi, Neslin, & Gedenk, 2001)	1.3	9/13	0.69			
It is important to me to get the best price for the products I buy. 1 Strongly disagree 2 Disagree 3 Neutral 4 Agree 5 Strongly agree	(Buckley, Cowan, & McCarthy, 2007)	1.3	9/14	0.64			
It is more important to choose food products for their nutritional value rather than their taste. 1 Strongly disagree 2 Disagree 3 Neutral 4 Agree 5 Strongly agree	(Scholderer, Brunsø, Bredahl, & Grunert, 2004)	1.3	9/14	0.64			
It is important to me that the food I eat on a typical day keeps me healthy. 1 Strongly disagree 2 Disagree 3 Neutral 4 Agree 5 Strongly agree	(Steptoe, Pollard, & Wardle, 1995)	1.3	9/14	0.64			
I compare product information labels to decide which brand to buy. 1 Strongly disagree 2 Disagree 3 Neutral 4 Agree 5 Strongly agree	(Scholderer, Brunsø, Bredahl, & Grunert, 2004)	1.3	8/13	0.62			
When cooking, I first and foremost consider the taste. 1 Strongly disagree 2 Disagree 3 Neutral 4 Agree 5 Strongly agree	(Scholderer, Brunsø, Bredahl, & Grunert, 2004)	1.3	8/13	0.62			
Do you consider nutrition, calories, and fat content when selecting menu items in fast-food restaurants. 1 Never 2 Rarely 3 Sometimes 4 Usually 5 Always	(Hwang & Cranage, 2010)	1.3	8/14	0.57			

Item	source	Round one[1] component	score	cvi	Round two component	score	CVI
How easy do you believe it is to determine the influence of external factors (social media, trends, promotional messages) on your food intake? 1 Very difficult 2 Difficult 3 Neutral 4 Easy 5 Very easy	(Palumbo et al., 2017)	1.3	8/14	0.57			
I find the taste of food products important. 1 Strongly disagree 2 Disagree 3 Neutral 4 Agree 5 Strongly agree	(Scholderer, Brunsø, Bredahl, & Grunert, 2004)	1.3	8/14	0.57			
I compare labels to select the most nutritious food. 1 Strongly disagree 2 Disagree 3 Neutral 4 Agree 5 Strongly agree	(Scholderer, Brunsø, Bredahl, & Grunert, 2004)	1.3	7/13	0.54			
It is important to me that the food I eat on a typical day can be cooked very simply. 1 Strongly disagree 2 Disagree 3 Neutral 4 Agree 5 Strongly agree	(Steptoe, Pollard, & Wardle, 1995)	1.3	7/13	0.54			
When deciding what foods to buy from food stores and restaurants, how often do you consider calories? 1 Never 2 Rarely 3 Sometimes 4 Usually 5 Always	(Brissette, Lowenfels, Noble, & Spicer, 2013)	1.3	7/14	0.50			
How easy do you believe it is to assess the value of the foods you intend to purchase and consume? 1 Very difficult 2 Difficult 3 Neutral 4 Easy 5 Very easy	(Palumbo et al., 2017)	1.3	6/13	0.46			
It is important to me that the food I eat on a typical day contains a lot of vitamins and minerals. 1 Strongly disagree 2 Disagree 3 Neutral 4 Agree 5 Strongly agree	(Steptoe, Pollard, & Wardle, 1995)	1.3	6/14	0.43			
It is important to me to know that I get quality for all my money. 1 Strongly disagree 2 Disagree 3 Neutral 4 Agree 5 Strongly agree	(Scholderer, Brunsø, Bredahl, & Grunert, 2004)	1.3	5/13	0.38			
I try to keep the amount of fat I eat at a healthy level. 1 Strongly disagree 2 Disagree 3 Neutral 4 Agree 5 Strongly agree	(Kearney et al., 2001)	1.3	4/12	0.33			
It is important to me that the food I eat on a typical day smells nice. 1 Strongly disagree 2 Disagree 3 Neutral 4 Agree 5 Strongly agree	(Steptoe, Pollard, & Wardle, 1995)	1.3	3/14	0.21			
COMPONENT 2.1 ACCESS FOOD THROUGH MULTIPLE SOURCES AND KNOW THE ADVANTAGES AND DISADVANTAGES OF THESE							
I compare prices between product variants in order to get the best value food. 1 Strongly disagree 2 Disagree 3 Neutral 4 Agree 5 Strongly agree	(Grunert, Brunsø, & Bisp, 1993)	1.3	10/14	0.71	2.1	51/64	0.80
I balance my intake of foods from restaurants, cafes, fast food outlets and what I make at home so that I can still have a healthy diet overall. 1 Strongly disagree 2 Disagree 3 Neutral 4 Agree 5 Strongly agree	Developed				2.1	48/65	0.74
I look for store specials and plan to take advantage of them when I go shopping. 1 Strongly disagree 2 Disagree 3 Neutral 4 Agree 5 Strongly agree	(Scholderer, Brunsø, Bredahl, & Grunert, 2004)	1.3	11/14	0.79	2.1	45/63	0.71
I balance my intake of foods from restaurants, cafes, fast food outlets and what I make at home so that I can still manage the amount of money I spend on food. 1 Strongly disagree 2 Disagree 3 Neutral 4 Agree 5 Strongly agree	Developed				2.1	45/66	0.68
I balance my intake of foods from restaurants, cafes, fast food outlets and what I make at home so that I can make the most of the convenience that each of these offers. 1 Strongly disagree 2 Disagree 3 Neutral 4 Agree 5 Strongly agree	Developed				2.1	39/66	0.59
Do you find it easy to determine which shops to go to for foods you need to make meals? 1 Very difficult 2 Difficult 3 Neutral 4 Easy 5 Very easy	(Dunn, Jayaratne, Baughman, & Levine, 2014)	2.1	12/15	0.80	2.1	38/66	0.58
Is price of food/value for money/specials an important influencer when deciding what to buy to eat outside the home? 1 Never 2 Rarely 3 Sometimes 4 Usually 5 Always	(NatCen Social Research, 2008)	2.1	10/15	0.67			
How easy do you believe it is to find information about the type of social and environmental impacts (sustainability, fairness ...) related to your eating habits? 1 Very difficult 2 Difficult 3 Neutral 4 Easy 5 Very easy	(Palumbo et al., 2017)	2.1	10/15	0.67			
Do you buy cheaper cuts of meat to save money? 1 Never 2 Rarely 3 Sometimes 4 Usually 5 Always	(Lavelle et al., 2017)	2.1	9/15	0.60			

Item	source	Round one[1]			Round two		
		component	score	cvi	component	score	CVI
Is quality or freshness of food an important influencer when deciding what to buy to eat outside the home? 1 Never 2 Rarely 3 Sometimes 4 Usually 5 Always	(NatCen Social Research, 2008)	2.1	9/15	0.60			
How easy do you believe it is to decide to follow a diet that meets your nutritional needs and at the same time takes care of the environment? 1 Very difficult 2 Difficult 3 Neutral 4 Easy 5 Very easy	(Palumbo et al., 2017)	2.1	9/15	0.60			
How easy do you believe it is to assess the impacts of environmental degradation on the quality of the food you use daily? 1 Very difficult 2 Difficult 3 Neutral 4 Easy 5 Very easy	(Palumbo et al., 2017)	2.1	7/12	0.58			
Is convenience/speed an important influencer when deciding what to buy to eat outside the home? 1 Never 2 Rarely 3 Sometimes 4 Usually 5 Always	(NatCen Social Research, 2008)	2.1	8/14	0.57			
Is taste of food an important influencer when deciding what to buy to eat outside the home? 1 Never 2 Rarely 3 Sometimes 4 Usually 5 Always	(NatCen Social Research, 2008)	2.1	7/15	0.47			
How easy do you believe it is to find information on how to change your eating habits to contribute to local area development? 1 Very difficult 2 Difficult 3 Neutral 4 Easy 5 Very easy	(Palumbo et al., 2017)	2.1	7/15	0.47			
How easy do you believe it is to assess what eating habits have a greater impact (in economic and social terms) on the external environment? 1 Very difficult 2 Difficult 3 Neutral 4 Easy 5 Very easy	(Palumbo et al., 2017)	2.1	7/15	0.47			
How easy do you believe it is to change your eating choices that express significant impacts on economic and environmental sustainability of its life context? 1 Very difficult 2 Difficult 3 Neutral 4 Easy 5 Very easy	(Palumbo et al., 2017)	2.1	6/14	0.43			
COMPONENT 2.2 DETERMINE WHAT IS IN A FOOD PRODUCT, WHERE IT CAME FROM, HOW TO STORE IT AND HOW TO USE IT							
Do you use the nutritional label on food products to guide your purchases? 1 Never 2 Rarely 3 Sometimes 4 Usually 5 Always	(Palumbo et al., 2017)	2.2	14/15	0.93	2.2	57/59	0.97
Do you compare the calories, fat, sugar or salt content of different products? 1 Never 2 Rarely 3 Sometimes 4 Usually 5 Always	(Poelman et al., 2018)	2.2	13/15	0.87	2.2	48/56	0.86
To me product information is of high importance. I need to know what the product contains. 1 Strongly disagree 2 Disagree 3 Neutral 4 Agree 5 Strongly agree	(Scholderer, Brunsø, Bredahl, & Grunert, 2004)	2.2	10/13	0.77	2.2	52/62	0.84
How easy do you believe it is to find information about the nutritional properties of the foods you plan to buy? 1 Very difficult 2 Difficult 3 Neutral 4 Easy 5 Very easy	(Palumbo et al., 2017)	2.2	12/15	0.80	2.2	52/64	0.81
Do you read the storage and use-by information on food packets? 1 Never 2 Rarely 3 Sometimes 4 Usually 5 Always	(Lavelle et al., 2017)	2.3	10/14	0.71	2.2	47/61	0.77
I know the environmental impact of my eating habits. 1 Strongly disagree 2 Disagree 3 Neutral 4 Agree 5 Strongly agree	Developed				2.2	50/65	0.77
How easy do you believe it is to find information about how to prepare the food that you purchased? 1 Very difficult 2 Difficult 3 Neutral 4 Easy 5 Very easy	(Palumbo et al., 2017)	2.2	11/15	0.73	2.2	48/64	0.75
I understand the ethical practices behind how the food I eat was produced. 1 Strongly disagree 2 Disagree 3 Neutral 4 Agree 5 Strongly agree	Developed				2.2	47/63	0.75
Is the impact on the community where food comes from an important influencer on your choice of foods? 1 Never 2 Rarely 3 Sometimes 4 Usually 5 Always	(NatCen Social Research, 2008)	2.1	11/15	0.73	2.2	45/62	0.73
I can make an accurate judgement on the nutritional content of the food I've selected. 1 Strongly disagree 2 Disagree 3 Neutral 4 Agree 5 Strongly agree	Developed				2.2	46/63	0.73
How often do you pay attention to the country of origin indications on food products you buy? 1 Never 2 Rarely 3 Sometimes 4 Usually 5 Always	(Cerjak, Haas, Brunner, & Tomic, 2014)	2.2	14/15	0.93	2.2	46/64	0.72
How easy do you believe it is to assess the nutritional properties of pre-prepared food (for example at a restaurant or takeaway) that you intend to buy? 1 Very difficult 2 Difficult 3 Neutral 4 Easy 5 Very easy	(Palumbo et al., 2017)	2.2	12/15	0.80	2.2	45/64	0.70

Item	source	Round one[1]			Round two		
		component	score	cvi	component	score	CVI
Do you read the nutrition information on food labels? 1 Never 2 Rarely 3 Sometimes 4 Usually 5 Always	(Lavelle et al., 2017)	2.2	12/13	0.92			
I like food to be unprocessed, even if this means that it takes more effort to prepare and keep fresh. 1 Strongly disagree 2 Disagree 3 Neutral 4 Agree 5 Strongly agree	(NatCen Social Research, 2008)	2.2	11/15	0.73			
I buy food that is processed as it is easier to prepare and store. 1 Strongly disagree 2 Disagree 3 Neutral 4 Agree 5 Strongly agree	(NatCen Social Research, 2008)	2.2	10/15	0.67			
How easy do you believe it is to find the origin of a food and the consequent risk to your health? 1 Very difficult 2 Difficult 3 Neutral 4 Easy 5 Very easy	(Palumbo et al., 2017)	2.2	10/15	0.67			
How easy do you believe it is to use the information on the food to meet your nutritional needs? 1 Very difficult 2 Difficult 3 Neutral 4 Easy 5 Very easy	(Palumbo et al., 2017)	2.2	10/15	0.67			
Do you check nutrition fact labels of products for calories, fat, sugar or salt content? 1 Never 2 Rarely 3 Sometimes 4 Usually 5 Always	(Poelman et al., 2018)	2.2	10/15	0.67			
How easy do you believe it is to consider how your food choices impact on environmental sustainability? 1 Very difficult 2 Difficult 3 Neutral 4 Easy 5 Very easy	(Palumbo et al., 2017)	2.1	9/15	0.60			
I understand the different methods of preservation and use of food. 1 Strongly disagree 2 Disagree 3 Neutral 4 Agree 5 Strongly agree	(Palumbo et al., 2017)	2.2	9/15	0.60			
How concerned are you about pesticide and fertiliser residues in food? 1 Not concerned 2 Not very concerned 3 Neutral 4 Concerned 5 Very concerned	(Worsley, Wang, & Burton, 2015)	2.2	8/15	0.53			
Do you believe it is easy to source information and digital resources to obtain greater and more reliable information on foods? 1 Very difficult 2 Difficult 3 Neutral 4 Easy 5 Very easy	(Palumbo et al., 2017)	2.2	7/15	0.47			
I like to know what I am buying, so I often ask questions in stores where I shop for food. 1 Strongly disagree 2 Disagree 3 Neutral 4 Agree 5 Strongly agree	(Grunert, Brunsø, & Bisp, 1993)	2.2	6/14	0.43			
How concerned are you about microbiological contamination of food products? 1 Not concerned 2 Not very concerned 3 Neutral 4 Concerned 5 Very concerned	(Worsley, Wang, & Burton, 2015)	2.2	6/14	0.43			
How concerned are you about routine use of animal antibiotics to promote growth of farm animals? 1 Not concerned 2 Not very concerned 3 Neutral 4 Concerned 5 Very concerned	(Worsley, Wang, & Burton, 2015)	2.2	5/15	0.33			
How easy do you believe it is to evaluate the convenience of the products that you intend to buy with respect to similar foodstuffs or alternatives? 1 Very difficult 2 Difficult 3 Neutral 4 Easy 5 Very easy	(Palumbo et al., 2017)	2.2	4/15	0.27			
How easy do you believe it is to understand why food additives or other substances are added to some foods to improve the commercial characteristics? 1 Very difficult 2 Difficult 3 Neutral 4 Easy 5 Very easy	(Palumbo et al., 2017)	2.2	3/15	0.20			
2.3 JUDGE THE QUALITY OF FOOD							
I can accurately judge the quality of fresh food so that it meets my expectations. 1 Strongly disagree 2 Disagree 3 Neutral 4 Agree 5 Strongly agree	Developed				2.3	52/63	0.83
Do you read the best-before date on food? 1 Never 2 Rarely 3 Sometimes 4 Usually 5 Always	(Lavelle et al., 2017)	2.3	13/15	0.87	2.3	50/64	0.78
I can accurately judge the quality of food I eat when I am out (restaurant, café) so that it meets my expectations. 1 Strongly disagree 2 Disagree 3 Neutral 4 Agree 5 Strongly agree	Developed				2.3	45/59	0.76
Do you buy food in season to save money? 1 Never 2 Rarely 3 Sometimes 4 Usually 5 Always	(Lavelle et al., 2017)	2.1	11/15	0.73	2.3	48/63	0.76
I can accurately judge the quality of processed or convenience food so that it meets my expectations. 1 Strongly disagree 2 Disagree 3 Neutral 4 Agree 5 Strongly agre	Developed				2.3	46/61	0.75
Are you able to see, smell or feel the quality of fresh foods? For example, of meat, fish or fruit 1 Never 2 Rarely 3 Sometimes 4 Usually 5 Always	(Poelman et al., 2018)	2.3	10/12	0.83	2.3	43/65	0.66

Item	source	Round one[1]			Round two		
		component	score	cvi	component	score	CVI
Quality means that the product is nutritious. 1 Strongly disagree 2 Disagree 3 Neutral 4 Agree 5 Strongly agree	(Chamhuri & Batt, 2015)	2.3	10/15	0.67			
I get confused over what's supposed to be healthy and what isn't. (The Health and Social Care Information Centre, 2009) 1 Strongly disagree 2 Disagree 3 Neutral 4 Agree 5 Strongly agree	(The Health and Social Care Information Centre, 2009)	2.3	9/14	0.64			
Quality means that the product is safe to eat. 1 Strongly disagree 2 Disagree 3 Neutral 4 Agree 5 Strongly agree	(Chamhuri & Batt, 2015)	2.3	9/15	0.60			
Quality means that the product will support my health. 1 Strongly disagree 2 Disagree 3 Neutral 4 Agree 5 Strongly agree	Developed	2.3	9/15	0.60			
Quality means that the product is free from antibiotics/growth promotants. 1 Strongly disagree 2 Disagree 3 Neutral 4 Agree 5 Strongly agree	(Chamhuri & Batt, 2015)	2.3	8/14	0.57			
Quality means that the product is fresh. 1 Strongly disagree 2 Disagree 3 Neutral 4 Agree 5 Strongly agree	(Chamhuri & Batt, 2015)	2.3	8/15	0.53			
Quality means that the product is free from pests and diseases. 1 Strongly disagree 2 Disagree 3 Neutral 4 Agree 5 Strongly agree	(Chamhuri & Batt, 2015)	2.3	7/14	0.50			
Quality means that the product will taste good. 1 Strongly disagree 2 Disagree 3 Neutral 4 Agree 5 Strongly agree	(Chamhuri & Batt, 2015)	2.3	7/14	0.50			
Quality means that the product will have a long shelf life. 1 Strongly disagree 2 Disagree 3 Neutral 4 Agree 5 Strongly agree	(Chamhuri & Batt, 2015)	2.3	6/14	0.43			
Quality means that the product is free from chemical residues. 1 Strongly disagree 2 Disagree 3 Neutral 4 Agree 5 Strongly agree	(Chamhuri & Batt, 2015)	2.3	6/15	0.40			
Quality means that I will not be disappointed when I eat the product. 1 Strongly disagree 2 Disagree 3 Neutral 4 Agree 5 Strongly agree	(Chamhuri & Batt, 2015)	2.3	5/13	0.38			
How easy do you believe it is to assess the food preservation status to verify the quality before consumption? (Palumbo et al., 2017) 1 Very difficult 2 Difficult 3 Neutral 4 Easy 5 Very easy	(Palumbo et al., 2017)	2.3	5/15	0.33			
Quality means the product is convenient. 1 Strongly disagree 2 Disagree 3 Neutral 4 Agree 5 Strongly agree	Developed	2.3	3/13	0.23			
How easy do you believe it is to actively participate in associations or interest groups to promote greater knowledge on food quality? (Palumbo et al., 2017) 1 Very difficult 2 Difficult 3 Neutral 4 Easy 5 Very easy	(Palumbo et al., 2017)	2.3	3/15	0.20			
3.1 MAKE A GOOD TASTING MEAL FROM WHATEVER FOOD IS AVAILABLE. THIS INCLUDES BEING ABLE TO PREPARE COMMONLY AVAILABLE FOODS, EFFICIENTLY USE COMMON PIECES OF KITCHEN EQUIPMENT AND HAVING A SUFFICIENT REPERTOIRE OF SKILLS TO ADAPT RECIPES (WRITTEN OR UNWRITTEN) TO EXPERIMENT WITH FOOD OR INGREDIENTS							
When lacking ingredients, do you find it easy to make changes to recipes without compromising the results?	(Palumbo et al., 2017)	3.1	12/12	1.00	3.1	59/63	0.94
How would you rate your skills in being able to cook from basic ingredients? 1 Very limited 2 Limited 3 Basic 4 Good 5 Very good	(Barton, Wrieden, & Anderson, 2011)	3.1	14/14	1.00	3.1	50/54	0.93
Are you able to cook from basic ingredients? 1 Never 2 Rarely 3 Sometimes 4 Usually 5 Always	(Garcia et al., 2014)	3.1	12/14	0.86	3.1	43/50	0.86
I am able to cook healthy foods for my family on a budget. 1 Strongly disagree 2 Disagree 3 Neutral 4 Agree 5 Strongly agree	(Pinard, Uvena, Quam, Smith, & Yaroch, 2015),	1.1	15/16	0.94	3.1	53/58	0.91
Are you able to prepare fresh vegetables in different ways? For example, cooking, steaming or stir frying or in different dishes 1 Never 2 Rarely 3 Sometimes 4 Usually 5 Always	(Poelman et al., 2018)	3.1	13/14	0.93	3.1	52/57	0.91
I am able to prepare a meal without a recipe. 1 Strongly disagree 2 Disagree 3 Neutral 4 Agree 5 Strongly agree	(Hartmann, Dohle, & Siegrist, 2013)	3.1	13/13	1.00	3.1	56/62	0.90

Item	source	Round one[1] component	score	cvi	Round two component	score	CVI
How confident are you at following recipes when cooking? 1 Not at all confident 2 Not very confident 3 Neutral 4 Confident 5 Extremely confident 	(Lavelle et al., 2017)	3.1	11/13	0.85	3.1	49/56	0.88
I feel I don't have the skills to cook (healthy) meals for my family. 1 Strongly disagree 2 Disagree 3 Neutral 4 Agree 5 Strongly agree	(Ramirez, 2015)	3.1	12/14	0.86	3.1	55/63	0.87
How good are you at each cooking task?					3.1	48/58	0.83
1. Handling pans on a stove	(Thonney & Bisogni, 2006)				3.1	41/57	0.72
2. Using an oven	(Thonney & Bisogni, 2006)				3.1	48/58	0.83
3. Steaming food (where the food doesn't touch the water but gets cooked by the steam)	(Lavelle et al., 2017)				3.1	45/56	0.80
4. Boiling or simmering food (cooking it in a pan of hot, boiling/bubbling water)	(Lavelle et al., 2017)				3.1	49/57	0.86
5. Stewing food (cooking it for a long time (usually more than an hour) in a liquid or sauce at a medium heat, not boiling) e.g. beef stew.	(Lavelle et al., 2017)				3.1	46/58	0.79
6. Roasting food in the oven, for example raw meat/chicken, fish, vegetables etc.	(Lavelle et al., 2017)				3.1	47/57	0.82
7. Frying/stir-frying/sautéing food in a frying pan/wok with oil or fat using the hob/gas rings/hot plates	(Lavelle et al., 2017)				3.1	48/58	0.83
8. Microwaving food (not drinks/liquid) including heating ready-meals	(Lavelle et al., 2017)				3.1	42/58	0.72
9. Cooking a piece of raw or frozen meat/chicken/fish, (not processed or partially-prepared)	(Thomas & Irwin, 2011)				3.1	52/58	0.88
10. Choosing a spice or herb that goes well with the food I am cooking	(Thomas & Irwin, 2011)				3.1	42/57	0.74
11. Baking muffins or cake using a pre-packaged mix	(Thomas & Irwin, 2011)		10/12	0.83	3.1	32/57	0.56
12. Baking muffins or cake from scratch with a recipe 1 Very limited 2 Limited 3 Basic 4 Good 5 Very good	(Thomas & Irwin, 2011)	3.1				39/58	0.67
Do you use leftovers to create another meal? 1 Never 2 Rarely 3 Sometimes 4 Usually 5 Always	(Lavelle et al., 2017)	3.1	11/14	0.79	3.1	51/63	0.81
I have a lot of knowledge about how to cook using different methods. 1 Strongly disagree 2 Disagree 3 Neutral 4 Agree 5 Strongly agree	(Burton, Reid, Worsley, & Mavondo, 2017)	3.1	11/13	0.85	3.1	46/58	0.79
Are you able to prepare fresh fish in different ways? For example, grilling, pan frying or stewing, or in different dishes 1 Never 2 Rarely 3 Sometimes 4 Usually 5 Always	(Poelman et al., 2018)	3.1	13/14	0.93	3.1	42/53	0.79
How would you rate your skills in planning a quick, healthy meal using only foods already in your home, and then preparing these foods so they can be served all together within 1 hour or less? 1 Very limited 2 Limited 3 Basic 4 Good 5 Very good	(Thomas & Irwin, 2011)	3.1	13/14	0.93	3.1	44/56	0.79
Are you able to prepare a meal using fresh ingredients? So, without pre-packed and processed foods? 1 Never 2 Rarely 3 Sometimes 4 Usually 5 Always	(Poelman et al., 2018)	3.1	13/14	0.93	3.1	46/60	0.77
How would you rate your skills in coordinating the preparation and cooking of a few food dishes at the same time so you can serve them all together for a meal? 1 Very limited 2 Limited 3 Basic 4 Good 5 Very good	(Thomas & Irwin, 2011)	3.1	12/14	0.86	3.1	41/54	0.76
Knowing how to cook is important to me. 1 Strongly disagree 2 Disagree 3 Neutral 4 Agree 5 Strongly agree	(Levy & Auld, 2004)	1.1	13/15	0.87	3.1	46/63	0.73
How good are you at each preparation task?	(Lavelle et al., 2017)				3.1	41/57	0.72
1. Using knife skills in the kitchen	(Condrasky et al., 2011)					45/56	0.80
2. Using measuring cups and spoons	(Thonney & Bisogni, 2006)					47/58	0.81
3. Using a grater	(Thonney & Bisogni, 2006)					36/56	0.64
4. Using a colander	(Thonney & Bisogni, 2006)					33/58	0.57
5. Kneading dough	(Thonney & Bisogni, 2006)	3.1	11/12	0.92		30/57	0.53

Item	source	Round one[1]			Round two		
		component	score	cvi	component	score	CVI
6. Chopping, mixing and stirring foods, for example chopping vegetables, dicing an onion, cubing meat, mixing and stirring food together in a pot/bowl	(Lavelle et al., 2017)					48/58	0.83
7. Peeling, chopping or slicing vegetables or fruit	(Thomas & Irwin, 2011)					49/58	0.84
8. Freezing vegetables or fruit, from raw to bagged in my home freezer	(Thomas & Irwin, 2011)					41/58	0.71
9.Blending foods to make them smooth, like soups or sauces (using a whisk/blender/food processor etc.) 1 Very limited 2 Limited 3 Basic 4 Good 5 Very good	(Lavelle et al., 2017)					40/57	0.70
Do you prepare or cook a healthy meal with only few ingredients on hand? 1 Never 2 Rarely 3 Sometimes 4 Usually 5 Always	(Lavelle et al., 2017)	3.1	12/13	0.92	3.1	43/61	0.70
I often believe that what I cook will turn out well. 1 Strongly disagree 2 Disagree 3 Neutral 4 Agree 5 Strongly agree	(Flego et al., 2013)	3.1	10/13	0.77	3.1	40/61	0.66
Do you prepare or cook a meal with limited time? 1 Never 2 Rarely 3 Sometimes 4 Usually 5 Always	(Lavelle et al., 2017)	3.1	13/14	0.93	3.1	37/61	0.61
I do not like to cook because it takes too much time. 1 Strongly disagree 2 Disagree 3 Neutral 4 Agree 5 Strongly agree	(Condrasky, Williams, Catalano, & Griffin, 2011)	1.1	12/15	0.80	3.1	45/62	0.73
How often do you prepare a meal? 1 Never 2 Less than once a week 3 1-3 times a week 4 4-6 times a week 5 Daily	(Byrd-Bredbenner, 2005)	3.1	11/13	0.85			
Do you find it difficult to prepare a meal with more than five fresh ingredients? 1 Never 2 Rarely 3 Sometimes 4 Usually 5 Always	(Poelman et al., 2018)	3.1	11/14	0.79			
Do you believe it is easy to follow recipes for the preparation of particular dishes? 1 Very difficult 2 Difficult 3 Neutral 4 Easy 5 Very easy	(Palumbo et al., 2017)	3.1	10/14	0.71			
In a normal week, how often do you prepare and cook a main meal from basic ingredients, for example making Shepherd's Pie starting with raw mince and potatoes? 1 Never 2 Less than once a week 3 1-3 times a week 4 4-6 times a week 5 Daily	(Barton et al., 2011)	3.1	9/13	0.69			
I consider my cooking skills as sufficient. 1 Strongly disagree 2 Disagree 3 Neutral 4 Agree 5 Strongly agree	(Hartmann et al., 2013)	3.1	8/12	0.67			
I often find that I buy ingredients, use them once, then leave them in the cupboard and never use them again. 1 Strongly disagree 2 Disagree 3 Neutral 4 Agree 5 Strongly agree	(Buckley, Cowan, & McCarthy, 2007)	3.1	9/14	0.64			
I find that I often have to throw away ingredients when cooking a meal from scratch. 1 Strongly disagree 2 Disagree 3 Neutral 4 Agree 5 Strongly agree	(Buckley, Cowan, & McCarthy, 2007)	3.1	9/14	0.64			
How good are you at using the same healthy ingredient in more than one meal? 1 Very limited 2 Limited 3 Basic 4 Good 5 Very good	(Pinard, Uvena, Quam, Smith, & Yaroch, 2015)	3.1	9/14	0.64			
I have the skills to prepare meals using:<br>1. Vegetables<br>2. Red meat<br>3. Chicken<br>4. White fish (e.g. cod, haddock, plaice)<br>5. Oily fish (e.g. herring, mackerel, salmon)<br>6. Pulses (e.g. bean, lentils)<br>7. Pasta<br>8. Rice (not rice pudding)<br>9. Potatoes (not chips)<br>10. Fresh green vegetables (e.g. cabbage, broccoli, spinach)<br>11. Root vegetables (e.g. carrot, parsnips, swede) 1 Never 2 Rarely 3 Sometimes 4 Usually 5 Always	(Winkler & Turrell, 2010)	3.1	7/13	0.54			

Item	source	Round one[1]			Round two		
		component	score	cvi	component	score	CVI
3.2 APPLY BASIC PRINCIPLES OF SAFE HYGIENE AND HANDLING							
Do you wash your hands before you start handling food? 1 Never 2 Rarely 3 Sometimes 4 Usually 5 Always	(Jevšnik, Hlebec, & Raspor, 2008)	3.2	11/12	0.92	3.2	55/57	0.96
Do you wash your hands after handling raw meat, poultry or fish? 1 Never 2 Rarely 3 Sometimes 4 Usually 5 Always	(Jevšnik, Hlebec, & Raspor, 2008)	3.2	11/13	0.85	3.2	52/54	0.96
Do you use the same knife for cutting raw and then cooked meat? 1 Never 2 Rarely 3 Sometimes 4 Usually 5 Always	(Jevšnik, Hlebec, & Raspor, 2008)	3.2	10/13	0.77	3.2	48/52	0.92
Do you wash fruit and vegetables that don't need to be peeled before eating them? 1 Never 2 Rarely 3 Sometimes 4 Usually 5 Always	(Barton et al., 2011)	3.2	10/14	0.71	3.2	51/57	0.89
I clean the kitchen surfaces before meals are prepared 1 Never 2 Rarely 3 Sometimes 4 Usually 5 Always	(Sanlier & Konaklioglu, 2012)	3.2	11/12	0.92	3.2	52/59	0.88
I know how to store meats and dairy. 1 Strongly disagree 2 Disagree 3 Neutral 4 Agree 5 Strongly agree	(Levy & Auld, 2004)				3.2	52/59	0.88
I clean the kitchen surfaces before and after meals are prepared 1 Never 2 Rarely 3 Sometimes 4 Usually 5 Always	(Sanlier & Konaklioglu, 2012)				3.2	52/59	0.88
Do you follow the instructions for storage on packaged foods? 1 Never 2 Rarely 3 Sometimes 4 Usually 5 Always	(Barton et al., 2011)	3.2	11/14	0.79	3.2	51/59	0.86
Once I unfreeze meat, I don't refreeze it again. 1 Never 2 Rarely 3 Sometimes 4 Usually 5 Always	(Sanlier & Konaklioglu, 2012)	3.2	12/14	0.86	3.2	47/57	0.82
I know how to store perishable foods (fruits, vegetables, meats, dairy). 1 Strongly disagree 2 Disagree 3 Neutral 4 Agree 5 Strongly agree	(Levy & Auld, 2004)	3.2	10/14	0.71	3.2	48/60	0.80
I keep raw and cooked meals separated. 1 Never 2 Rarely 3 Sometimes 4 Usually 5 Always	(Sanlier & Konaklioglu, 2012)	3.2	11/14	0.79	3.2	49/56	0.88
If you have a sore on the back of your hand, should you prepare food for other people? 1 No, you should not prepare food until the sore heals. 2 Yes, if you bandage the sore and wear a glove. 3 Yes, if you wear a glove. 4 Yes, if you put a bandage on it. 5 Yes, if it isn't infected.	(Byrd-Bredbenner et al., 2007)	3.2	9/14	0.64			
The best way to keep from getting food poisoning from fresh fruit and vegetables it to wash them with? 1 Cool running water 2 An antibacterial sponge 3 Antibacterial soap 4 Hot water 5 Regular soap	(Byrd-Bredbenner et al., 2007)	3.2	9/14	0.64			
I keep foods waiting in room temperature until it cools down. 1 Never 2 Rarely 3 Sometimes 4 Usually 5 Always	(Sanlier & Konaklioglu, 2012)	3.2	9/14	0.64			
I don't touch cooked meals after I touch raw meals. 1 Never 2 Rarely 3 Sometimes 4 Usually 5 Always	(Sanlier & Konaklioglu, 2012)	3.2	9/14	0.64			
I put foods into the fridge within two hours of buying them. 1 Never 2 Rarely 3 Sometimes 4 Usually 5 Always	(Sanlier & Konaklioglu, 2012)	3.2	9/14	0.64			
If a family member is going to be several hours late for a hot meal, how should you store the meal to keep it safe until this person is ready to eat it? 1 Do not know 2 Store it in a warm oven until the person is ready to eat it 3 Store it in a cool oven until the person is ready to eat it 4 Store it in on the kitchen counter until the person is ready to eat it 5 Store it in the refrigerator and reheat it when the person is ready to eat it	(Byrd-Bredbenner et al., 2007)	3.2	8/13	0.62			
How easy do you believe it is to look up information on food safety and hygiene practices? 1 Very difficult 2 Difficult 3 Neutral 4 Easy 5 Very easy	(Palumbo et al., 2017)	3.2	8/13	0.62			
I defrost meat on the kitchen counter. 1 Never 2 Rarely 3 Sometimes 4 Usually 5 Always	(Sanlier & Konaklioglu, 2012)	3.2	8/13	0.62			
Do you check that food is piping hot when re-heating? 1 Never 2 Rarely 3 Sometimes 4 Usually 5 Always	(Barton et al., 2011)	3.2	8/14	0.57			
After you have used a cutting board to slice raw meat, chicken, or fish and need to cut other foods, which of these is the best way to prevent food poisoning? 1 Set the cutting board aside and use a different	(Byrd-Bredbenner et al., 2007)	3.2	8/14	0.57			

Item	source	Round one[1] component	score	cvi	Round two component	score	CVI
cutting board to cut other foods 2 Wash the cutting board with hot soapy water and rinse 3 Turn the board over and use the other side 4 Rinse the cutting board under very hot water 5 Wipe the cutting board off with a paper towel							
Salmonella bacteria can cause food poisoning. How can a food be made safe if it has salmonella in it? 1 Do not know 2 The food cannot be made safe 3 Freeze it for at least 3 days 4 Wash it under extremely hot running water 5 Cook it thoroughly	(Byrd-Bredbenner et al., 2007)	3.2	8/14	0.57			
You may contaminate the next food you touch with salmonella bacteria if you don't wash your hands after touching: 1 Do not know 2 Raw chicken 3 Raw beef 4 Raw sprouts and lettuce 5 Raw pork	(Byrd-Bredbenner et al., 2007)	3.2	8/14	0.57			
What is the recommended freezer temperature for preventing food poisoning? 1 Do not know 2 32 °F (0 °C) 3 24 °F (−4 °C) 4 18 °F (−8 °C) 5 0 °F (−18 °C)	(Byrd-Bredbenner et al., 2007)	3.2	8/14	0.57			
What is the maximum temperature refrigerators should be to preserve the safety of foods? 1 60 °F (16 °C) 2 45 °F (7 °C) 3 40 °F (4 °C) 4 25 °F (−4 °C) 5 0 °F (−18 °C)	(Byrd-Bredbenner et al., 2007)	3.2	8/14	0.57			
To prevent food poisoning, which of these individuals should not prepare food for other people? 1 None of these individuals 2 A person with diarrhea 3 A person with HIV 4 A person who smokes 5 A person with unexplained itching	(Byrd-Bredbenner et al., 2007)	3.2	7/14	0.50			
To prevent food poisoning, how often should the kitchen sink drain in your home be sanitized? 1 Do not know 2 Only when food is going to be thawed or washed in the sink 3 Monthly 4 Weekly 5 Daily	(Byrd-Bredbenner et al., 2007)	3.2	7/14	0.50			
For ground beef to be safe to eat, it needs to be cooked until its internal temperature reaches? 1 Do not know 2 250 °F (121 °C) 3 160 °F (71 °C) 4 125 °F (52 °C) 5 90 °F (32 °C)	(Byrd-Bredbenner et al., 2007)	3.2	6/13	0.46			
All foods are considered safe when cooked to an internal temperature of? 1 Do not know 2 165 °F (74 °C) 3 150 °F (66 °C) 4 140 °F (60 °C) 5 130 °F (54 °C)	(Byrd-Bredbenner et al., 2007)	3.2	6/13	0.46			
I taste the milk whether it is safe or not, instead of looking at its' pull date. 1 Never 2 Rarely 3 Sometimes 4 Usually 5 Always	(Sanlier & Konaklioglu, 2012)	3.2	6/14	0.43			
Do you eat food past its 'use-by' date? 1 Never 2 Rarely 3 Sometimes 4 Usually 5 Always	(Barton et al., 2011)	3.2	5/14	0.36			
I break and use dirty eggs carefully and I do not wash them. 1 Never 2 Rarely 3 Sometimes 4 Usually 5 Always	(Sanlier & Konaklioglu, 2012)	3.2	5/14	0.36			
People should be especially careful about not eating raw seafood if they have? 1 Cancer 2 HIV Infection 3 Diabetes 4 Any of these diseases 5 Do not know	(Byrd-Bredbenner et al., 2007)	3.2	4/13	0.31			
Listeria bacteria is most likely with which food? 1 Do not know 2 Raw eggs or poultry 3 Deli meats 4 Raw or undercooked beef 5 Home canned food	(Byrd-Bredbenner et al., 2007)	3.2	4/13	0.31			
4.1. UNDERSTAND FOOD HAS AN IMPACT ON PERSONAL WELLBEING							
I understand what foods to eat to prevent diet related chronic disease (such as heart disease). 1 Strongly disagree 2 Disagree 3 Neutral 4 Agree 5 Strongly agree	Developed	4.1	12/15	0.80	4.1	48/53	0.91
The types of food I eat affects how I feel 1 Strongly disagree 2 Disagree 3 Neutral 4 Agree 5 Strongly agree	(Bauer, Larson, Nelson, Story, & Neumark-Sztainer, 2009)				4.1	55/61	0.90
The types of food I eat affects my health 1 Strongly disagree 2 Disagree 3 Neutral 4 Agree 5 Strongly agree	(Bauer, Larson, Nelson, Story, & Neumark-Sztainer, 2009)				4.1	49/55	0.89
The types of food I eat affects my weight 1 Strongly disagree 2 Disagree 3 Neutral 4 Agree 5 Strongly agree	(Bauer, Larson, Nelson, Story, & Neumark-Sztainer, 2009)	4.1	13/15	0.87	4.1	49/61	0.80

Item	source	Round one[1]			Round two		
		component	score	cvi	component	score	CVI
The types of food I eat affects how I look 1 Strongly disagree 2 Disagree 3 Neutral 4 Agree 5 Strongly agree	(Bauer, Larson, Nelson, Story, & Neumark-Sztainer, 2009)				4.1	32/57	0.56
How easy do you believe it is to find information on foods for the prevention or management of a disease or condition? 1 Very difficult 2 Difficult 3 Neutral 4 Easy 5 Very easy	(Palumbo et al., 2017)	4.1	12/15	0.80	4.1	42/58	0.72
In general, how much control do you have over your health status? 1 None 2 Very little 3 Some 4 Quite a bit 5 Very much	(Worsley, Wang, & Burton, 2015)	4.1	10/14	0.71	4.1	38/58	0.66
How easy do you believe it is to use the food/nutritional recommendations to improve your state of health? 1 Very difficult 2 Difficult 3 Neutral 4 Easy 5 Very easy	(Palumbo et al., 2017)	4.1	11/15	0.73	4.1	48/58	0.83
How easy do you believe it is to decide whether to consume foods that help prevent the onset of chronic conditions such as overweight, obesity, diabetes, high blood pressure? 1 Very difficult 2 Difficult 3 Neutral 4 Easy 5 Very easy	(Palumbo et al., 2017)	4.1	10/15	0.67			
How easy do you believe it is to understand the information on the beneficial health effects of certain types of foods? 1 Very difficult 2 Difficult 3 Neutral 4 Easy 5 Very easy	(Palumbo et al., 2017)	4.1	9/14	0.64			
How easy do you believe it is to evaluate the pros and cons of a diet that you intend to follow? 1 Very difficult 2 Difficult 3 Neutral 4 Easy 5 Very easy	(Palumbo et al., 2017)	4.1	9/14	0.64			
If I eat a healthy diet I can greatly reduce my chance of getting heart disease. 1 Strongly disagree 2 Disagree 3 Neutral 4 Agree 5 Strongly agree	(Food and Drug Administration, 2016)	4.1	9/14	0.64			
How easy do you believe it is to find information about how to prepare food to be taken during a state of illness or disease? 1 Very difficult 2 Difficult 3 Neutral 4 Easy 5 Very easy	(Palumbo et al., 2017)	4.1	9/15	0.60			
How easy do you believe it is to understand the useful recommendations to follow a healthy eating pattern and limit the consumption of saturated fats? 1 Very difficult 2 Difficult 3 Neutral 4 Easy 5 Very easy	(Palumbo et al., 2017)	4.1	9/15	0.60			
How easy do you believe it is to change your eating habits that may worsen your health condition? 1 Very difficult 2 Difficult 3 Neutral 4 Easy 5 Very easy	(Palumbo et al., 2017)	4.1	9/15	0.60			
If I eat a healthy diet I can greatly reduce my chance of getting cancer. 1 Strongly disagree 2 Disagree 3 Neutral 4 Agree 5 Strongly agree	(Food and Drug Administration, 2016)	4.1	9/15	0.60			
What are the main factors that influence what you eat on a regular basis?HealthEffect on physical appearance Not important Slightly important Important Very important Extremely important	(Vilaro et al., 2017)	4.1	7/12	0.58			
How easy do you believe it is to search information on how to prevent or treat conditions such as overweight by modifying your eating habits? 1 Very difficult 2 Difficult 3 Neutral 4 Easy 5 Very easy	(Palumbo et al., 2017)	4.1	8/15	0.53			
How easy do you believe it is to determine whether the nutritional information you collected is suitable for your specific health needs? 1 Very difficult 2 Difficult 3 Neutral 4 Easy 5 Very easy	(Palumbo et al., 2017)	4.1	8/15	0.53			
How easy do you believe it is to understand which diet is most suitable in case of illness or during a disease? 1 Very difficult 2 Difficult 3 Neutral 4 Easy 5 Very easy	(Palumbo et al., 2017)	4.1	6/15	0.40			
How easy do you believe it is to learn about food consumption strategies that favor the individual psycho-physical well-being? 1 Very difficult 2 Difficult 3 Neutral 4 Easy 5 Very easy	(Palumbo et al., 2017)	4.1	6/15	0.40			
How easy do you believe it is to find information on how to handle critical situations such as an uncontrollable hunger or desire to consume alcohol? 1 Very difficult 2 Difficult 3 Neutral 4 Easy 5 Very easy	(Palumbo et al., 2017)	4.1	5/15	0.33			
How easy do you believe it is to find information on how and when you can consume high-calorie foods? 1 Very difficult 2 Difficult 3 Neutral 4 Easy 5 Very easy	(Palumbo et al., 2017)	4.1	3/15	0.20			
How easy do you believe it is to decide if you consume foods not commonly used, but with high antioxidant properties? 1 Very difficult 2 Difficult 3 Neutral 4 Easy 5 Very easy	(Palumbo et al., 2017)	4.1	3/15	0.20			

Item	source	Round one[1]			Round two		
		component	score	cvi	component	score	CVI
4.2 DEMONSTRATE SELF-AWARENESS OF THE NEED TO PERSONALLY BALANCE FOOD INTAKE. THIS INCLUDES KNOWING FOODS TO INCLUDE FOR GOOD HEALTH, FOOD TO RESTRICT FOR GOOD HEALTH, AND APPROPRIATE PORTION SIZE AND FREQUENCY							
I think about nutrition when I choose what I eat. 1 Never 2 Rarely 3 Sometimes 4 Usually 5 Always	(Stotts & Lohse, 2007)	4.2	12/14	0.86	4.2	50/54	0.93
I make a conscious effort to try to follow a healthy diet. 1 Strongly disagree 2 Disagree 3 Neutral 4 Agree 5 Strongly agree	(Kearney et al., 2001)	1.3	10/14	0.71	4.2	48/52	0.92
Do health professionals recommend that people should be eating more, the same amount, or less of the following foods? 1. Fruit 2. Food and drinks with added sugar 3. Vegetables 4. Fatty foods 5. Processed red meat 6. Wholegrain 7. Salty foods 8. Water 1 Not sure 2 Less 3 Same 4 More	(Kliemann, Wardle, Johnson, & Croker, 2016)	4.2	10/14	0.71	4.2	48/53	0.91
How many servings of fruit and vegetables per day do you think you should eat as a minimum? (One serving could be, for example, an apple or a handful of chopped carrots) 2 3 4 5 ore more Not sure	(Kliemann et al., 2016)	4.2	12/14	0.86	4.2	51/58	0.88
I know what foods to eat to keep me healthy. 1 Strongly disagree 2 Disagree 3 Neutral 4 Agree 5 Strongly agree	Developed	4.1	14/15	0.93	4.2	50/58	0.86
When deciding what to feed your family, how often do you think about healthy food choices? 1 Never 2 Rarely 3 Sometimes 4 Usually 5 Always	(Dollahite, Pijai, Scott-Pierce, Parker, & Trochim, 2014)	1.3	11/14	0.79	4.2	50/58	0.86
Which of these types of fats do health professionals recommend that people should eat less of?Unsaturated fatsTrans fatsSaturated fats 1 Not sure 2 Eat less 3 Not eat less	(Kliemann et al., 2016)	4.2	11/13	0.85	4.2	49/58	0.84
c) Do you balance meals based on nutrition advice on what is healthy? 1 Never 2 Rarely 3 Sometimes 4 Usually 5 Always	(Lavelle et al., 2017)	1.3	10/14	0.71		40/50	0.80
How easy do you believe it is to understand the food/nutritional recommendations provided by your doctor or dietitian? 1 Very difficult 2 Difficult 3 Neutral 4 Easy 5 Very easy	(Palumbo et al., 2017)	4.1	10/14	0.71	4.2	45/57	0.79
Do you eat the total amount of a package or container of crisps, candies or cookies at once? 1 Never 2 Rarely 3 Sometimes 4 Usually 5 Always	(Poelman et al., 2018)	4.2	10/13	0.77	4.2	26/58	0.45
Imagine that you are at a place where you see and smell tasty foods. Are you able to resist the temptation of buying this? For example, at the train, the petrol station or at the bakery? 1 Never 2 Rarely 3 Sometimes 4 Usually 5 Always	(Poelman et al., 2018)	4.2	10/14	0.71	4.2	26/59	0.44
Do you eat vegetable as snacks? 1 Never 2 Rarely 3 Sometimes 4 Usually 5 Always	(Poelman et al., 2018)	4.2	13/14	0.93			
Do you eat fruit as a snack? 1 Never 2 Rarely 3 Sometimes 4 Usually 5 Always	(Poelman et al., 2018)	4.2	13/14	0.93			
Are you able to say 'no' to tasty snacks if you want to? For example, birthday treats or finger foods 1 Never 2 Rarely 3 Sometimes 4 Usually 5 Always	(Poelman et al., 2018)	4.2	8/12	0.67			
Are you able to eat healthy if the situation deviates from a regular situation? For example, when you have unexpected guests or experience time pressure 1 Never 2 Rarely 3 Sometimes 4 Usually 5 Always	(Poelman et al., 2018)	4.2	8/13	0.62			
Do you choose foods that are in line with your mood? For example, if you are sad or annoyed? 1 Never 2 Rarely 3 Sometimes 4 Usually 5 Always	(Poelman et al., 2018)	4.2	8/14	0.57			
4.3 JOIN IN AND EAT IN A SOCIAL WAY							
How often do you eat together at home with others? 1 Never 2 Rarely 3 Sometimes 4 Usually 5 Always	(Herbert et al., 2014)	4.3	11/14	0.79	4.3	52/60	0.87
Mealtime is a time for talking with others, it is about more than just eating food. 1 Strongly disagree 2 Disagree 3 Neutral 4 Agree 5 Strongly agree	(Woodruff & Kirby, 2013)	4.3	11/13	0.85	4.3	54/58	0.93
I find that dining with friends is an important part of my social life. 1 Strongly disagree 2 Disagree 3 Neutral 4 Agree 5 Strongly agree	(Grunert, Brunsø, & Bisp, 1993)	4.3	10/13	0.77	4.3	53/60	0.88
Do you find it important to eat at the dinner table if you are eating with others? 1 Never 2 Rarely 3 Sometimes 4 Usually 5 Always	(Poelman et al., 2018)	4.3	14/14	1.00	4.3	52/59	0.88

Item	source	Round one[1]			Round two		
		component	score	cvi	component	score	CVI
I eat on the go. 1 Never 2 Rarely 3 Sometimes 4 Usually 5 Always	(Crawford, Ball, Mishra, Salmon, & Timperio, 2007)	4.3	12/14	0.86	4.3	49/58	0.84
Are you involved in other activities while eating? For example, reading, working, using ipad/iphone or watching television?	(Poelman et al., 2018)	4.3	13/13	1.00	4.3	46/55	0.84
Eating brings people together in an enjoyable way. 1 Strongly disagree 2 Disagree 3 Neutral 4 Agree 5 Strongly agree	neumark (Neumark-Sztainer, Larson, Fulkerson, Eisenberg, & Story, 2010)	4.3	12/14	0.86	4.3	47/59	0.80
My eating habits and behaviours make it hard for me to join in with others when I'm eating. 1 Strongly disagree 2 Disagree 3 Neutral 4 Agree 5 Strongly agree	Developed				4.3	41/52	0.79
I sit at a table to eat my meals. 1 Never 2 Rarely 3 Sometimes 4 Usually 5 Always	Developed	4.3	12/14	0.86	4.3	46/60	0.77
In my household people often have their meals at different times. 1 Never 2 Rarely 3 Sometimes 4 Usually 5 Always	(Mallinson, Russell, & Barker, 2016)	4.3	12/14	0.86	4.3	46/60	0.77
It is important that I eat at least one meal a day with others. 1 Strongly disagree 2 Disagree 3 Neutral 4 Agree 5 Strongly agree	(Woodruff & Kirby, 2013)	4.3	12/14	0.86	4.3	38/50	0.76
I eat meals/snacks while working or studying. 1 Never 2 Rarely 3 Sometimes 4 Usually 5 Always	(Crawford, Ball, Mishra, Salmon, & Timperio, 2007)	4.3	10/13	0.77	4.3	37/49	0.76
Do you find it important to eat dinner at the same time if you are with others? 1 Never 2 Rarely 3 Sometimes 4 Usually 5 Always	(Poelman et al., 2018)	4.3	12/14	0.86	4.3	38/51	0.75
My eating habits exclude me from social groups. 1 Strongly disagree 2 Disagree 3 Neutral 4 Agree 5 Strongly agree	Developed				4.3	33/49	0.67
I feel uncomfortable eating with others due to my limited knowledge of eating etiquette. 1 Strongly disagree 2 Disagree 3 Neutral 4 Agree 5 Strongly agree	Developed				4.3	29/53	0.55
I avoid eating with others due to my limited knowledge of eating etiquette. 1 Strongly disagree 2 Disagree 3 Neutral 4 Agree 5 Strongly agree	Developed				4.3	22/48	0.46
I don't know what is expected of me when I'm sharing a meal with others. 1 Strongly disagree 2 Disagree 3 Neutral 4 Agree 5 Strongly agree	Developed				4.3	23/51	0.45
I don't know what to do when I'm sharing a meal with others. 1 Strongly disagree 2 Disagree 3 Neutral 4 Agree 5 Strongly agree	Developed				4.3	22/50	0.44
I prefer eating with people than alone. 1 Never 2 Rarely 3 Sometimes 4 Usually 5 Always		4.3	10/14	0.71	4.3	39/50	0.78
When I serve dinner to friends, the most important thing is that we are together. (Scholderer et al., 2004) 1 Strongly disagree 2 Disagree 3 Neutral 4 Agree 5 Strongly agree	(Scholderer, Brunsø, Bredahl, & Grunert, 2004)	4.3	8/14	0.57			
We often get together with friends to enjoy an easy-to-cook, casual dinner. (Grunert et al., 1993) 1 Strongly disagree 2 Disagree 3 Neutral 4 Agree 5 Strongly agree	(Grunert, Brunsø, & Bisp, 1993)	4.3	8/14	0.57			

Ailawadi, K. L., Neslin, S. A., & Gedenk, K. (2001). Pursuing the Value-Conscious Consumer: Store Brands versus National Brand Promotions. *J Mark, 65*(1), 71-89. doi:10.1509/jmkg.65.1.71.18132

Barton, K. L., Wrieden, W. L., & Anderson, A. S. (2011). Validity and reliability of a short questionnaire for assessing the impact of cooking skills interventions. *Journal of Human Nutrition and Dietetics, 24*(6), 588-595. doi:10.1111/j.1365-277X.2011.01180.x

Bauer, K. W., Larson, N. I., Nelson, M. C., Story, M., & Neumark-Sztainer, D. (2009). Socio-environmental, personal and behavioural predictors of fast-food intake among adolescents. *Public Health Nutrition, 12*(10), 1767-1774. doi:10.1017/S1368980008004394

Bell, R., & Marshall, D. W. (2003). The construct of food involvement in behavioral research: scale development and validation. *APPETITE, 40*(3), 235-244. doi:10.1016/S0195-6663(03)00009-6

Brissette, I., Lowenfels, A., Noble, C., & Spicer, D. (2013). Predictors of total calories purchased at fast-food restaurants: Restaurant characteristics, calorie awareness, and use of calorie information. *J Nutr Educ Behav, 45*(5), 404-411. doi:10.1016/j.jneb.2013.01.019

Buckley, M., Cowan, C., & McCarthy, M. (2007). The convenience food market in Great Britain: Convenience food lifestyle (CFL) segments. *APPETITE, 49*(3), 600-617. doi:10.1016/j.appet.2007.03.226

Burton, M., Reid, M., Worsley, A., & Mavondo, F. (2017). Food skills confidence and household gatekeepers' dietary practices. *APPETITE, 108*, 183-190. doi:10.1016/j.appet.2016.09.033

Byrd-Bredbenner, C. (2005). Food Preparation Knowledge and Confidence of Young Adults. *Journal of Nutrition in Recipe & Menu Development, 3*(3-4), 37-50. doi:10.1300/J071v03n03_04

Byrd-Bredbenner, C., Wheatley, V., Schaffner, D., Bruhn, C., Blalock, L., & Maurer, J. (2007). Development and implementation of a food safety knowledge instrument: Food science education research. *Journal of Food Science Education, 6*(3), 46-55. doi:10.1111/j.1541-4329.2007.00029.x

Cerjak, M., Haas, R., Brunner, F., & Tomic, M. (2014). What motivates consumers to buy traditional food products? Evidence from Croatia and Austria using word association and laddering interviews. *Br Food J., 116*(11), 1726-1747. doi:10.1108/BFJ-02-2014-0090

Chamhuri, N., & Batt, P. J. (2015). Consumer perceptions of food quality in Malaysia. *Br Food J., 117*(3), 1168-1187. doi:10.1108/BFJ-08-2013-0235

Condrasky, M. D., Williams, J. E., Catalano, P. M., & Griffin, S. F. (2011). Development of psychosocial scales for evaluating the impact of a culinary nutrition education program on cooking and healthful eating. *J Nutr Educ Behav, 43*(6), 511-516. doi:10.1016/j.jneb.2010.09.013

Crawford, D., Ball, K., Mishra, G., Salmon, J., & Timperio, A. (2007). Which food-related behaviours are associated with healthier intakes of fruits and vegetables among women? *Public Health Nutrition, 10*(3), 256-265. doi:10.1017/S1368980007246798

Dollahite, J. S., Pijai, E. I., Scott-Pierce, M., Parker, C., & Trochim, W. (2014). A Randomized Controlled Trial of a Community-Based Nutrition Education Program for Low-Income Parents. *J Nutr Educ Behav, 46*(2), 102-109. doi:10.1016/j.jneb.2013.09.004

Dunn, C., Jayaratne, K. S. U., Baughman, K., & Levine, K. (2014). Teaching Basic Cooking Skills: Evaluation of the North Carolina Extension "Cook Smart, Eat Smart" Program. *J Fam Consum Sci, 106*(1), 39-46.

Flego, A., Herbert, J., Gibbs, L., Swinburn, B., Keating, C., Waters, E., & Moodie, M. (2013). Methods for the evaluation of the Jamie Oliver Ministry of Food program, Australia. *BMC Public Health, 13*(1), 411. doi:10.1186/1471-2458-13-411

Food and Drug Administration. (2016). *2014 FDA Health and Diet Survey*. Retrieved from https://www.fda.gov/downloads/Food/FoodScienceResearch/ConsumerBehaviorResearch/UCM497251.pdf

Garcia, A. L., Vargas, E., Lam, P. S., Shennan, D. B., Smith, F., & Parrett, A. (2014). Evaluation of a cooking skills programme in parents of young children - a longitudinal study. *Public Health Nutrition, 17*(5), 1013-1021. doi:10.1017/S1368980013000165

Grunert, K. G., Brunsø, K., & Bisp, S. (1993). *Food-related life style: Development of a cross-culturally valid instrument for market surveillance*. MAPP working paper no 12. https://pure.au.dk/ws/files/88/wp12.pdf

Hartmann, C., Dohle, S., & Siegrist, M. (2013). Importance of cooking skills for balanced food choices. *APPETITE, 65*, 125-131. doi:10.1016/j.appet.2013.01.016

Herbert, J., Flego, A., Gibbs, L., Waters, E., Swinburn, B., Reynolds, J., & Moodie, M. (2014). Wider impacts of a 10-week community cooking skills program - Jamie's Ministry of Food, Australia. *BMC Public Health, 14*(1), 1161. doi:10.1186/1471-2458-14-1161

Hwang, J., & Cranage, D. (2010). Customer health perceptions of selected fast-food restaurants according to their nutritional knowledge and health consciousness. *Journal of Foodservice Business Research, 13*(2), 68-84. doi:10.1080/15378021003781174

Jevšnik, M., Hlebec, V., & Raspor, P. (2008). Consumers' awareness of food safety from shopping to eating. *Food Control, 19*(8), 737-745. doi:10.1016/j.foodcont.2007.07.017

Kearney, J. M., Gibney, M. J., Livingstone, B. E., Robson, P. J., Kiely, M., & Harrington, K. (2001). Attitudes towards and beliefs about nutrition and health among a random sample of adults in the Republic of Ireland and Northern Ireland. *Public Health Nutrition, 4*(5a), 1117-1126. doi:10.1079/PHN2001193

Kliemann, N., Wardle, J., Johnson, F., & Croker, H. (2016). Reliability and validity of a revised version of the General Nutrition Knowledge Questionnaire. *European Journal of Clinical Nutrition, 70*(10), 1174-1180. doi:10.1038/ejcn.2016.87

Lavelle, F., McGowan, L., Hollywood, L., Surgenor, D., McCloat, A., Mooney, E., . . . Dean, M. (2017). The development and validation of measures to assess cooking skills and food skills. *Int J Behav Nutr Phys Act., 14*, 118. doi:10.1186/s12966-017-0575-y

Levy, J., & Auld, G. (2004). Cooking Classes Outperform Cooking Demonstrations for College Sophomores. *J Nutr Educ Behav, 36*(4), 197-203. doi:10.1016/S1499-4046(06)60234-0

Mallinson, L. J., Russell, J. M., & Barker, M. E. (2016). Attitudes and behaviour towards convenience food and food waste in the United Kingdom. *APPETITE, 103*, 17-28. doi:10.1016/j.appet.2016.03.017

NatCen Social Research. (2008). British Social Attitudes Survey. Retrieved from http://bsa.natcen.ac.uk/media/38990/bsa-26-annotated-questionnaires-2008.pdf

Neumark-Sztainer, D., Larson, N. I., Fulkerson, J. A., Eisenberg, M. E., & Story, M. (2010). Family meals and adolescents: what have we learned from Project EAT (Eating Among Teens)? *Public Health Nutrition, 13*(7), 1113-1121. doi:10.1017/S1368980010000169

Palumbo, R., Annarumma, C., Adinolfi, P., Vezzosi, S., Troiano, E., Catinello, G., & Manna, R. (2017). Crafting and applying a tool to assess food literacy: Findings from a pilot study. *Trends Food Sci Technol., 67*, 173-182. doi:10.1016/j.tifs.2017.07.002

Pinard, C. A., Uvena, L. M., Quam, J. B., Smith, T. M., & Yaroch, A. L. (2015). Development and testing of a revised Cooking Matters for Adults Survey. *American Journal of Health Behavior, 39*(6), 866-873. doi:10.5993/AJHB.39.6.14

Poelman, M. P., Dijkstra, S. C., Sponselee, H., Kamphuis, C. B. M., Battjes-Fries, M. C. E., Gillebaart, M., & Seidell, J. C. (2018). Towards the measurement of food literacy with respect to healthy eating: the development and validation of the self perceived food literacy scale among an adult sample in the Netherlands. *Int J Behav Nutr Phys Act, 15*(1), 54. doi:10.1186/s12966-018-0687-z

Ramirez, E. (2015). *Development and implementation of the generations eating together through cooking (G.E.T.T. Cooking) curriculum and its effects on an inter-generational population: A pilot study.* (Degree Doctor of Philosophy), Clemson University, Clemson, South Carolina, USA.

Sanlier, N., & Konaklioglu, E. (2012). Food safety knowledge, attitude and food handling practices of students. *Br Food J, 114*(4), 469-480. doi:10.1108/00070701211219504
Scholderer, J., Brunsø, K., Bredahl, L., & Grunert, K. G. (2004). Cross-cultural validity of the food-related lifestyles instrument (FRL) within Western Europe. *APPETITE, 42*(2), 197-211. doi:10.1016/j.appet.2003.11.005
Steptoe, A., Pollard, T. M., & Wardle, J. (1995). Development of a Measure of the Motives Underlying the Selection of Food: the Food Choice Questionnaire. *APPETITE, 25*(3), 267-284. doi:10.1006/appe.1995.0061
Stotts, J. L., & Lohse, B. (2007). Reliability of the ecSatter Inventory as a Tool to Measure Eating Competence. *J Nutr Educ Behav, 39*(5, Supplement), S167-S170. doi:10.1016/j.jneb.2007.03.091
The Health and Social Care Information Centre. (2009). Health survey for England 2007, Healthy lifestyles: Knowledge, attitudes and behaviour. Retrieved from http://digital.nhs.uk/catalogue/PUB00415
Thomas, H. M., & Irwin, J. D. (2011). Cook It Up! A community-based cooking program for at-risk youth: Overview of a food literacy intervention. *BMC Research Notes, 4*(1), 495. doi:10.1186/1756-0500-4-495
Thonney, P. F., & Bisogni, C. A. (2006). Cooking Up Fun! A Youth Development Strategy that Promotes Independent Food Skills. *J Nutr Educ Behav, 38*(5), 321-323. doi:10.1016/j.jneb.2006.03.007
Vilaro, M. J., Zhou, W., Colby, S. E., Byrd-Bredbenner, C., Riggsbee, K., Olfert, M. D., . . . Mathews, A. E. (2017). Development and Preliminary Testing of the Food Choice Priorities Survey (FCPS): Assessing the Importance of Multiple Factors on College Students' Food Choices. *Evaluation & the Health Professions, 40*(4), 425-449. doi:10.1177/0163278717735872
Winkler, E., & Turrell, G. (2010). Confidence to Cook Vegetables and the Buying Habits of Australian Households. *Journal of the American Dietetic Association, 110*(5), S52-S61. doi:10.1016/j.jada.2010.03.007
Woodruff, S. J., & Kirby, A. R. (2013). The associations among family meal frequency, food preparation frequency, self-efficacy for cooking, and food preparation techniques in children and adolescents. *J Nutr Educ Behav, 45*(4), 296-303. doi:10.1016/j.jneb.2012.11.006
Worsley, A., Wang, W. C., & Burton, M. (2015). Food concerns and support for environmental food policies and purchasing. *APPETITE, 91*, 48-55. doi:10.1016/j.appet.2015.02.040
